# Identification of glucocorticoid-related molecular signature by whole blood methylome analysis

**DOI:** 10.1530/EJE-21-0907

**Published:** 2021-12-16

**Authors:** Roberta Armignacco, Anne Jouinot, Lucas Bouys, Amandine Septier, Thomas Lartigue, Mario Neou, Cassandra Gaspar, Karine Perlemoine, Leah Braun, Anna Riester, Fidéline Bonnet-Serrano, Anne Blanchard, Laurence Amar, Carla Scaroni, Filippo Ceccato, Gian Paolo Rossi, Tracy Ann Williams, Casper K Larsen, Stéphanie Allassonnière, Maria-Christina Zennaro, Felix Beuschlein, Martin Reincke, Jérôme Bertherat, Guillaume Assié

**Affiliations:** 1 Université de Paris, Institut Cochin, INSERM U1016, CNRS UMR8104, Paris, France; 2 ARAMIS Project-Team, Inria Paris, France; 3 CMAP, UMR 7641, CNRS, École polytechnique, I.P. Paris, France; 4 Sorbonne Université, Inserm, UMS Pass, Plateforme Post-génomique de la Pitié-Salpêtrière, P3S, Paris, France; 5 Medizinische Klinik und Poliklinik IV, Klinikum der Universität, Ludwig-Maximilians-Universität München, Munich, Germany; 6 Assistance Publique-Hôpitaux de Paris, Hôpital Cochin, Service d’Hormonologie, Paris, France; 7 Assistance Publique-Hôpitaux de Paris, Hôpital Européen Georges Pompidou, Centre d’Investigations Cliniques 9201, Paris, France; 8 Université de Paris, PARCC, INSERM, Paris, France; 9 Assistance Publique-Hôpitaux de Paris, Hôpital Européen Georges Pompidou, Unité Hypertension Artérielle, Paris, France; 10 UOC Endocrinologia, Dipartimento di Medicina DIMED, Azienda Ospedaliera-Università di Padova, Padua, Italy; 11 Clinica dell’Ipertensione Arteriosa, Department of Medicine-DIMED, University of Padua, Padua, Italy; 12 Division of Internal Medicine and Hypertension Unit, Department of Medical Sciences, University of Turin, Turin, Italy; 13 CRC, UMR S1138, Université de Paris, INSERM, Sorbonne Université, Paris, France; 14 Assistance Publique-Hôpitaux de Paris, Hôpital Européen Georges Pompidou, Service de Génétique, Paris, France; 15 Klinik für Endokrinologie, Diabetologie und Klinische Ernährung, UniversitätsSpital Zürich, Zürich, Switzerland; 16 Assistance Publique-Hôpitaux de Paris, Hôpital Cochin, Service d’Endocrinologie, Center for Rare Adrenal Diseases, Paris, France

## Abstract

**Objective:**

Cushing’s syndrome represents a state of excessive glucocorticoids related to glucocorticoid treatments or to endogenous hypercortisolism. Cushing’s syndrome is associated with high morbidity, with significant inter-individual variability. Likewise, adrenal insufficiency is a life-threatening condition of cortisol deprivation. Currently, hormone assays contribute to identify Cushing’s syndrome or adrenal insufficiency. However, no biomarker directly quantifies the biological glucocorticoid action. The aim of this study was to identify such markers.

**Design:**

We evaluated whole blood DNA methylome in 94 samples obtained from patients with different glucocorticoid states (Cushing’s syndrome, eucortisolism, adrenal insufficiency). We used an independent cohort of 91 samples for validation.

**Methods:**

Leukocyte DNA was obtained from whole blood samples. Methylome was determined using the Illumina methylation chip array (~850 000 CpG sites). Both unsupervised (principal component analysis) and supervised (Limma) methods were used to explore methylome profiles. A Lasso-penalized regression was used to select optimal discriminating features.

**Results:**

Whole blood methylation profile was able to discriminate samples by their glucocorticoid status: glucocorticoid excess was associated with DNA hypomethylation, recovering within months after Cushing’s syndrome correction. In Cushing’s syndrome, an enrichment in hypomethylated CpG sites was observed in the region of *FKBP5* gene locus. A methylation predictor of glucocorticoid excess was built on a training cohort and validated on two independent cohorts. Potential CpG sites associated with the risk for specific complications, such as glucocorticoid-related hypertension or osteoporosis, were identified, needing now to be confirmed on independent cohorts.

**Conclusions:**

Whole blood DNA methylome is dynamically impacted by glucocorticoids. This biomarker could contribute to better assessment of glucocorticoid action beyond hormone assays.

## Introduction

Cushing’s syndrome is a state of glucocorticoid excess related either to glucocorticoid treatment (exogenous Cushing’s syndrome) or to excessive secretion of adrenocortical glucocorticoids. While effective in suppressing inflammatory states (1), the prolonged use of administered glucocorticoids is associated with potentially serious adverse effects, restricting their widespread and chronic usage (2). Clinical consequences of systemic glucocorticoid treatment are mirrored by states of endogenous cortisol excess. While overt manifestations of Cushing’s syndrome are rare and most often related to pituitary adenomas (3), mild autonomous cortisol hypersecretion is more common and mostly caused by adrenal adenomas (4).

Cushing’s syndrome is associated with high morbidity and mortality, and impaired quality of life (5) through numerous systemic manifestations, including diabetes mellitus, hypertension, osteoporosis, cutaneous bruising, muscular atrophy, neuropsychiatric disorders, and immune deficiency (6). Duration and level of glucocorticoid excess are undisputedly the main determinants of Cushing’s syndrome severity. However, individual susceptibility highly affects the likelihood to develop each type of complication and modulates their severity (7).

On the other side of the clinical spectrum, adrenal insufficiency is a state of cortisol deprivation, inducing fatigue and acute decompensations of metabolism or electrolyte balance with potentially lethal outcome (8). Adrenal insufficiency can be caused by structural or functional damage of adrenal glands, pituitary, or hypothalamus, with autoimmunity-, tumour-, or treatment-related reasons being the most common causes.

Quantification of glucocorticoid action on peripheral tissues is difficult to assess by clinical means or on the basis of hormonal evaluations. Indeed, for exogenous glucocorticoid administration, pharmacokinetics may importantly influence the level of glucocorticoid excess, especially for low-dose systemic treatments or in case of local administration. For endogenous Cushing’s syndrome, increased morbidity and mortality related to mild autonomous cortisol excess are well demonstrated on the population level (9, 10) but cannot be estimated properly on an individual level using classical hormone assays. For patients with adrenal insufficiency, titration of glucocorticoid supplementation relies mainly on clinical assessment. This shortcoming underlines the need for specific biomarkers quantifying glucocorticoid action, with potential impact on diagnosis, treatment decision, and prediction of the individual risk for specific complications.

DNA methylation is a chemically stable yet dynamic biological hallmark, playing a key role in epigenetic regulation of gene expression in both health and disease (11). Several studies have suggested an association between hypothalamic–pituitary–adrenal axis dysregulation and specific blood DNA methylation profiles, particularly in post-traumatic stress disorders (12, 13, 14, 15, 16). In addition, the association between stress and DNA methylation has been explored for some targeted genes (17). Among them, methylation of *FKBP5* – encoding a co-chaperone of HSP90 protein involved in the regulation of glucocorticoid receptor activity (18) – and *NR3C1* – encoding the glucocorticoid receptor – are impacted by stress. Furthermore, a recent study showed a correlation between *FKBP5* expression and cortisol levels in patients with Cushing’s syndrome (19).

The present study explores the impact of glucocorticoids on leukocyte DNA methylation. Specifically, we analysed whole blood methylome in patients with endogenous Cushing’s syndrome, eucortisolism, or adrenal insufficiency, and we identified a methylome signature reflecting glucocorticoid excess.

## Subjects and methods

### Patients and samples

Ninety-four blood samples were collected from 47 patients with a confirmed diagnosis of endogenous Cushing’s syndrome. Patients were followed in two expert centres, Cochin hospital (APHP, Paris, France) and LMU hospital (Ludwig-Maximilians-University, Munich, Germany). Diagnostic criteria of Cushing’s syndrome included: increased 24-h urine-free cortisol, abnormal cortisol after 1 mg dexamethasone suppression, and altered circadian cortisol rhythm, following consensus guidelines (20).

Blood samples were collected either before correction of Cushing’s syndrome or at least 3 months after (3–41 months; median: 12 months). At the time of blood sampling, patients were classified as overt Cushing’s syndrome, mild Cushing’s syndrome, and eucortisolism or adrenal insufficiency, depending on clinical evaluation and hormone assays. Briefly, overt Cushing’s syndrome patients presented clinical signs and increased 24-h urine-free cortisol (>240 nmol/24 h), increased salivary midnight cortisol (>6 nmol/L), and insufficient cortisol suppression after 1 mg dexamethasone (>50 nmol/L). Mild Cushing’s syndrome patients lacked signs of clinically overt Cushing’s syndrome but had mild alterations of cortisol secretion, including either a slightly increased 24-h urine-free cortisol, or increased midnight salivary cortisol, or insufficient cortisol suppression after 1 mg dexamethasone. Adrenal insufficiency was based on low plasma morning cortisol (<160 nmol/L) and on insufficient response to corticotropin stimulation (<500 nmol/L). For two patients in eucortisolism, exact values were not available. Detailed hormone values for each sample are provided in Supplementary Table 1 (see section on supplementary materials given at the end of this article).

Signed informed consent for molecular analysis of blood samples and for access to clinical data was obtained from all patients, and the study was approved by a local Ethic Committee (for Cochin hospital: Comité de Protection de Personnes Ile de France 1, project 13495; for Munich: project 152-10).

Ninety-one additional samples were available and suitable for methylome analysis from patients enrolled in five specialized centres of the ENSAT-HT consortium (http://www.ensat-ht.eu). They included 26 patients with endogenous Cushing’s syndrome (20) and 65 healthy volunteers (eucortisolism) (Supplementary Table 2). Signed informed consent was obtained from all patients, and the study was approved by the Ethic Committee of each participating centre.

### Whole-genome DNA methylation measurement

Leukocyte DNA was extracted from EDTA blood samples, using the DNA Isolation kit for Mammalian Blood (Roche). DNA quality was assessed on a Genomic DNA ScreenTape system (Agilent) and quantified using a Qubit 3.0 Fluorometer (Thermofisher). DNA was treated by bisulphite and then hybridized to the Infinium MethylationEPIC BeadChip (Illumina; ~850 000 sites), starting from 500 ng of DNA. All experiments were performed following the manufacturer’s instructions at the P3S Post-Genomic Platform of Sorbonne University (Paris, France).

### Bioinformatics and statistics

All samples passed the quality controls provided by the Genome Studio software (v. 2011.1; Illumina). Data were exported as Intensity Data (IDAT) format and then processed using the minfi package (v. 1.32.0) (21) in R software environment (v. 3.6.3) (https://cran.r-project.org/).

Data were normalized using the stratified quantile normalization procedure implemented in the preprocessQuantile minfi function (22) and the methylation score for each CpG probe was extracted as a β-value. The ChAMP package (v. 2.16.1) was used to filter the probes (23). A total of 731 635 probes passed the following criteria: detection *P* -value < 0.01, presence of the targeted CpG, absence of frequent SNPs in the probe, single hybridization hit, and autosomal target.

The significant components of variation in the data set were assessed using the singular value decomposition method (SVD) for methylation data (24) and a detected batch effect (Slide) was corrected using the ComBat method (25), as implemented in the ChAMP package.

White blood cell count of subpopulations (neutrophils, lymphocytes B, lymphocytes T4, lymphocytes T8, lymphocytes NK, and monocytes) was estimated by the reference-based RefbaseEWAS method (26) implemented in the ChAMP package. To confirm the reliability of the inferred white blood cell counts, we compared the estimated and available measured proportions of both neutrophils and lymphocytes, obtaining high correlation (Pearson’s r = 0.81 and r = 0.87, respectively) (Supplementary Fig. 1 and Supplementary Table 3). Since neutrophils were the most represented cell type in all samples, and since the proportions of neutrophils and lymphocytes were negatively correlated (Pearson’s r = −0.97), the estimated proportion of neutrophils was chosen as the unique proxy reflecting variations in white blood cell count.

M-values, used for statistical analyses, were calculated from β-values (log_2_ ratio of the intensities of methylated vs unmethylated probes) using the lumi package (v. 2.36.0) (27).

Global data structure was assessed on β-values by principal component analysis (PCA), using all CpG probes. Probe variability was calculated on M-values as s.d. for each CpG site among samples. The most variable CpG probes (*n* = 52 727 with s.d. > 0.4) were selected for subsequent analyses.

Differentially methylated CpG sites were identified starting from the whole data set using the Limma package (v. 3.40.6) (28), including the estimated neutrophils count as covariate, and considering a Benjamin-Hochberg adjusted *P* -value < 0.05. Gene set enrichment analysis of genes associated with differentially methylated CpG sites was performed using the gometh method implemented in the missmethyl package (v. 1.18.0) (29), adjusting for the number of CpG sites associated to each gene (30). Differentially methylated regions were identified using the DMRcate package (v. 1.20.0) (31), comparing overt Cushing’s syndrome vs eucortisolism or adrenal insufficiency, and using estimated neutrophil’s count as covariate. Default parameters were applied to smooth the differential methylation signal and to define regions.

For predicting the glucocorticoid status from methylation, a training cohort of 60 samples was selected, randomly including 30 samples corresponding to overt Cushing’s syndrome with no anti-cortisolic treatment at the time of sampling and 30 samples corresponding to either eucortisolism or adrenal insufficiency after Cushing’s syndrome treatment. Remaining samples (*n* = 34) were used as a first validation cohort. CpG site selection was performed on the training cohort using a penalized Lasso regression on the most variable CpG probes (M-value s.d. > 0.4), with a ten-fold cross-validation, using the glmnet package (v. 4.0-2) (32). The predictive model, including 29 discriminating CpG sites, was assessed on the validation cohort, graphically using a PCA projection of samples based on the 29 CpGs methylation level and statistically using an ordinal logistic regression model including a 29-CpGs predictor, calculated by adding the 29 CpGs M-values weighted by their Lasso coefficients. Similarly, the performance of the 29-CpGs methylation predictor was tested on the ENSAT-HT cohort, a second independent validation cohort.

In order to identify potential CpG sites specifically associated to glucocorticoid-related complications (hypertension, diabetes, and osteoporosis), CpG site selection was performed on 47 Cushing’s samples, starting from the most variable CpG probes (M-value s.d. > 0.4), using a penalized Lasso regression with a ten-fold cross-validation. A methylation predictor for each model was calculated as described for the 29-CpGs predictor.

Quantitative variable comparisons between groups were performed using two-tailed *t*-test or Wilcoxon’s test, depending on variable distribution. Quantitative variable correlations were performed using Pearson’s test. Multivariate analysis was performed using a logistic regression model including the 29-CpGs methylation predictor and the estimated proportion of neutrophils as covariates. All tests were computed in R software environment.

## Results

### Cohort presentation

Ninety-four samples were collected from 47 patients with endogenous Cushing’s syndrome (Table 1 and Supplementary Table 1). Median age was 46 years (range: 17–73), with a female predominance (1.8–1). Patients with endogenous Cushing’s syndrome included Cushing’s disease (*n* = 39), benign adrenal Cushing’s syndrome (*n* = 7), and ectopic ACTH over-secretion (*n* = 1). Cushing’s syndrome-associated hypertension, diabetes, osteoporosis, and catabolism – that is, presence of either osteoporosis, osteopenia, muscle weakness, pigmented striae or other skin lesions – were present in 36 (77%), 16 (34%), 13 (28%), and 30 (64%) patients, respectively. Samples were collected at different time points during the course of the disease, thereby reflecting different states of glucocorticoid secretion: overt Cushing’s syndrome (*n* = 42), mild Cushing’s syndrome (*n* = 13), eucortisolism several months after Cushing’s syndrome treatment (*n* = 14), or adrenal insufficiency several months after Cushing’s syndrome treatment (*n* = 25). Samples were assigned either to training or to validation cohorts, as described in the ‘Materials and methods’ section.

**Table 1:** Characteristics of the samples tested. Cortisol values are provided as median values with ranges.

Glucocorticoid status	Normal range	Global cohort	Training cohort	Validation cohort	P*
Total number of samples		94	60	34	
Overt Cushing’s syndrome					
*n*		42	30	12	
Urinary free cortisol, nmol/24 h	<240	1163 (306–44 375)	1907 (306–44 375)	896 (329–3496)	0.021
Midnight salivary cortisol, nmol/L	<6	20 (6–194)	22 (6–194)	13 (6–97)	0.046
Plasma cortisol after 1 mg DST, nmol/L	<50	377 (74–1883)	400 (74–1883)	293 (110–822)	0.155
Mild Cushing’s syndrome					
*n*		13		13	
Urinary free cortisol, nmol/24 h	<240	213 (68–360)		213 (68–360)	
Midnight salivary cortisol, nmol/L	<6	10 (3–17)		10 (3–17)	
Plasma cortisol after 1 mg DST, nmol/L	<50	64 (32–215)		64 (32–215)	
Eucortisolism					
*n*		14	8	6	
Urinary-free cortisol, nmol/24 h	<240	188 (71–304)	125 (97–276)	207 (71–304)	0.690
Midnight salivary cortisol, nmol/L	<6	4 (1–11)	3 (1–5)	5 (2–11)	0.167
Plasma cortisol after 1 mg DST, nmol/L	<50	37 (25–48)	37 (30–48)	35 (25–44)	0.8
Adrenal insufficiency					
*n*		25	22	3	
Early morning plasma cortisol, nmol/L	160–500	83 (6–287)	82 (6–287)	97 (17–218)	0.645
Cortisol after ACTH stimulation, nmol/L	>500	276 (19–1322)	331 (19–1322)	91 (41–1092)	0.616

*Wilcoxon’s test comparing training and validation cohorts.

An additional independent cohort of 91 samples, part of the European ENSAT-HT consortium, was collected, including 26 patients with endogenous Cushing’s syndrome and 65 healthy volunteers (eucortisolism; Supplementary Table 2).

### Glucocorticoid levels impact blood methylome

**Figure 1:**
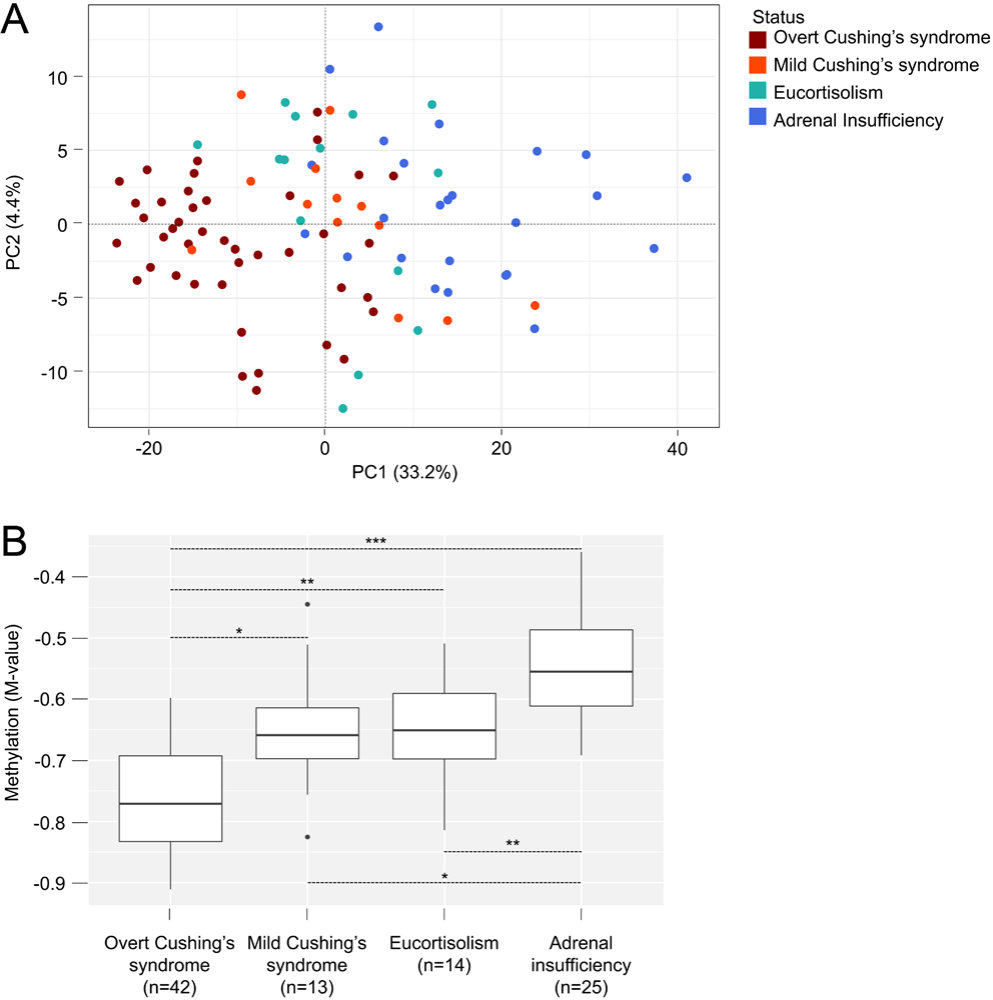
Glucocorticoid levels impact on whole blood DNA methylation. (A) Samples projection based on the two principle components (PC1, PC2) of unsupervized PCA performed on the whole data set (*n* = 731 635 CpG sites, *n*  = 94 samples). (B) Representation of global methylation (median M-value) relative to the most variable CpG sites (*n* = 52 727 with a M-value s.d. > 0.4) in the four groups. *P -value < 0.05, **P -value < 0.001, ***P -value < 10^−10^.

Whole blood DNA methylome was determined for the 94 samples, with 731 635 informative CpG sites in all samples. Unsupervized PCA showed a discrimination of samples according to their glucocorticoid status, with a specific profile of overt Cushing’s syndrome (Fig. 1A). This discrimination was mainly related to the global methylation level. Indeed, overt Cushing’s syndrome status was associated with overall decreased methylation among the most variable CpG sites (*t*-test *P* -value < 0.05 for 52 727 CpG sites; Fig. 1B). Another significant determinant was the white blood cell count variation (Supplementary Fig. 2), related to the well-established effect of glucocorticoids on white blood cell composition, inducing granulocytosis and lymphopenia (33, 34).

### Exploration of glucocorticoid-related blood methylome profile

**Figure 2:**
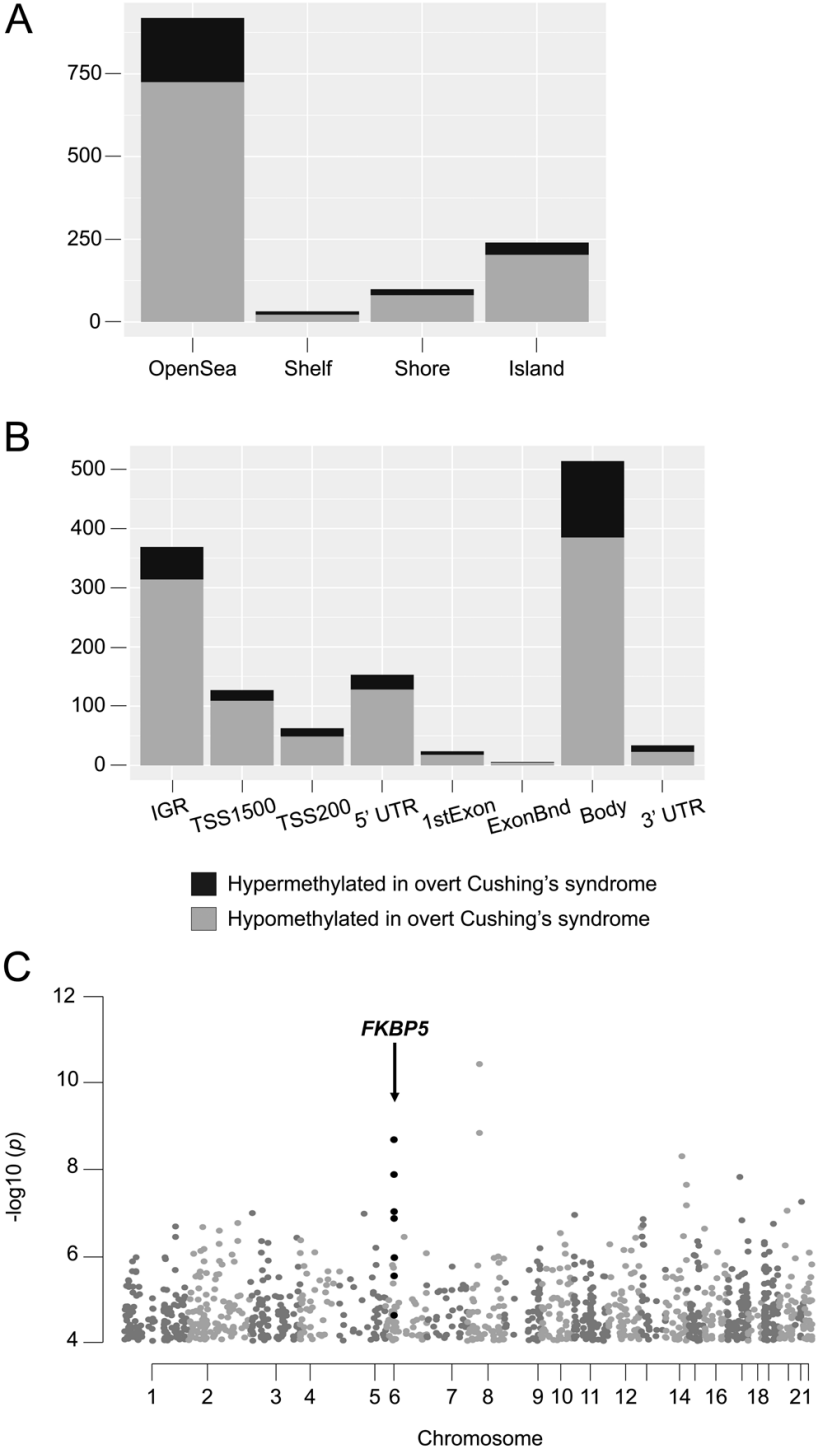
Distribution of differentially methylated CpG sites (overt Cushing’s syndrome vs eucortisolism: *n*  = 1290). (A) Distribution relative to genome CpG enrichment. (B) Distribution relative to gene locus structure. (C) Genomic distribution. Highlighted in black, the CpG sites located in the *FKBP5* gene locus on chromosome 6.

The specific effect of glucocorticoids on blood methylome was evaluated by comparing the methylation level in overt Cushing’s syndrome samples (*n* = 42) vs each of the other three groups individually – mild Cushing’s syndrome (*n* = 13), eucortisolism (*n* = 14), and adrenal insufficiency (*n* = 25) samples (Supplementary Table 4). The most significant difference was observed in the comparisons of overt Cushing’s syndrome vs eucortisolism (*n* = 1290 differentially methylated CpG sites) and overt Cushing’s syndrome vs adrenal insufficiency (*n* = 7120 differentially methylated CpG sites). Both comparisons showed a prevalence of hypomethylated CpG sites in overt Cushing’s syndrome (80 and 73%, respectively). Hypomethylated CpG sites were observed both in ‘Open Sea’ and ‘Island’ regions, showing the independence of glucocorticoid-related hypomethylation from CpG enrichment in the genome (Fig. 2A and Supplementary Fig. 3A). Glucocorticoid-related hypomethylation was not related to any specific gene locus structure either (Fig. 2B and Supplementary Fig. 3B).

GSEA of genes associated with the differentially methylated CpG sites in the two comparisons revealed an enrichment in immunity-related signalling pathways (Gene Ontology gene sets; FDR < 0.05), particularly those relating to neutrophils degranulation (Supplementary Tables 5 and 6).

**Figure 3:**
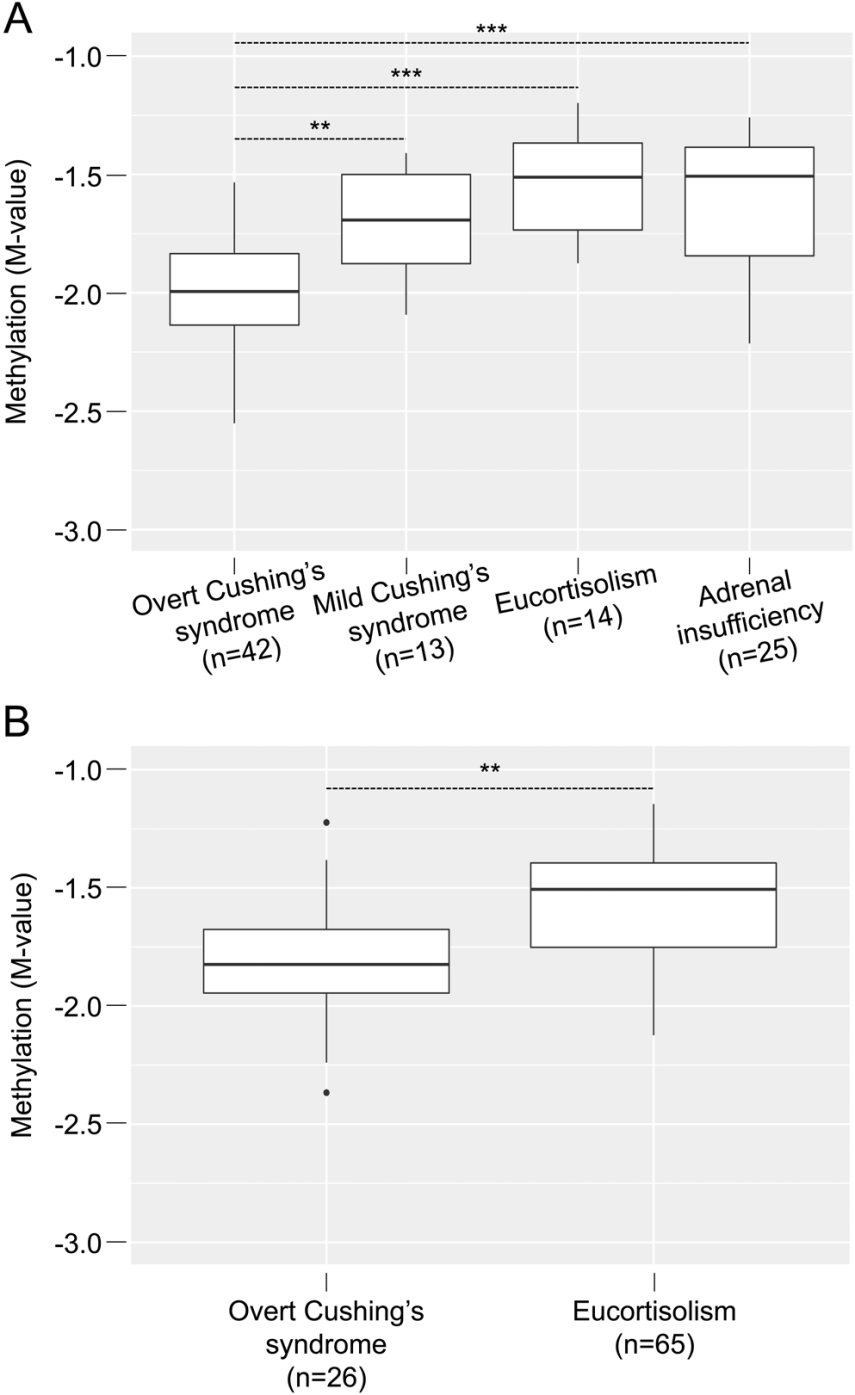
Methylation level of the *FKBP5* promoter region in Cushing’s syndrome samples. (A) Boxplot representation of the mean methylation (M-value) of the five CpG sites included in the differentially methylated region associated to the *FKBP5* gene promoter, in the principal cohort. (B) Boxplot representation of the methylation level of the same five CpG sites in the ENSAT-HT cohort. **P -value < 0.001, ***P -value < 10^−5^.

Differentially methylated CpG sites were distributed all along the genome. One gene locus was strongly enriched in differential CpG sites, on chromosome 6, corresponding to the *FKBP5* gene locus (Fig. 2C, Supplementary Fig. 3C and Supplementary Table 4). Beyond the analysis of individual CpG sites, a specific analysis of differentially methylated regions identified 99 and 418 differentially methylated regions in overt Cushing’s syndrome vs eucortisolism and vs adrenal insufficiency, respectively (Supplementary Tables 7 and 8). Again, one of the most significant differentially methylated regions associated with the *FKBP5* gene promoter (Stouffer’s Z-score < 0.001) (Supplementary Table 7). This *FKBP5* promoter region included 5 CpG sites, whose methylation level properly discriminated overt Cushing’s syndrome samples from all the others (*t*-test *P* -value < 0.05; Fig. 3A). This effect was tested on the ENSAT-HT cohort, confirming the lower methylation level of these *FKBP5*-associated CpG sites in Cushing’s syndrome (Fig. 3B). Since promoter methylation usually negatively correlates with gene expression (11), we measured *FKBP5* gene expression in a subset of 37 samples (14 overt Cushing’s syndrome, 10 mild Cushing’s syndrome, 7 eucortisolism, 6 adrenal insufficiency), for which whole blood RNA was available (Supplementary Methods). *FKBP5* expression was negatively correlated with the five *FKBP5* promoter-associated CpG sites (r = −0.55, *P* -value < 0.001), and positively with the 24-h urine-free cortisol (Pearson’s r = 0.62, *P* -value < 0.001), demonstrating the potential interest of using *FKBP5* expression as a biomarker of glucocorticoid excess.

**Figure 4:**
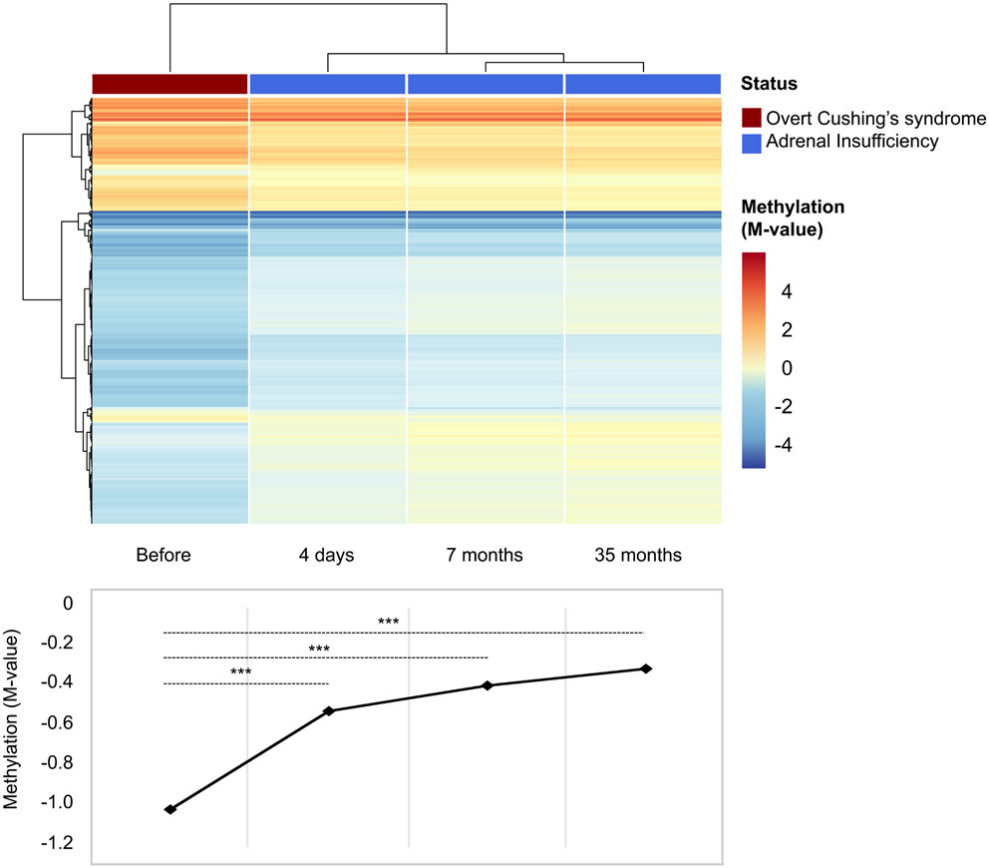
Kinetics of methylome modifications after normalization of glucocorticoid excess. Unsupervised clustering of four samples from patient P30, collected before and at three different time points after Cushing’s syndrome correction. The mean methylation (M-value) of the 7426 CpG sites differentially methylated in overt Cushing’s syndrome is provided below. ***P -value < 10^−15^.

We next explored the kinetics of glucocorticoid-related methylome modification by comparing the methylation profile of four different samples available for one of the patients (patient P30), collected before, 4 days, 7 months, and 35 months after Cushing’s syndrome correction. Hierarchical clustering of methylome profiles well discriminated the overt Cushing’s syndrome sample. During the months following Cushing’s syndrome correction, the three samples were properly ordered, showing a progressive overall re-increase of methylation (*t*-test *P* -value < 0.05; Fig. 4).

### Predicting glucocorticoid status by blood DNA methylation

**Figure 5:**
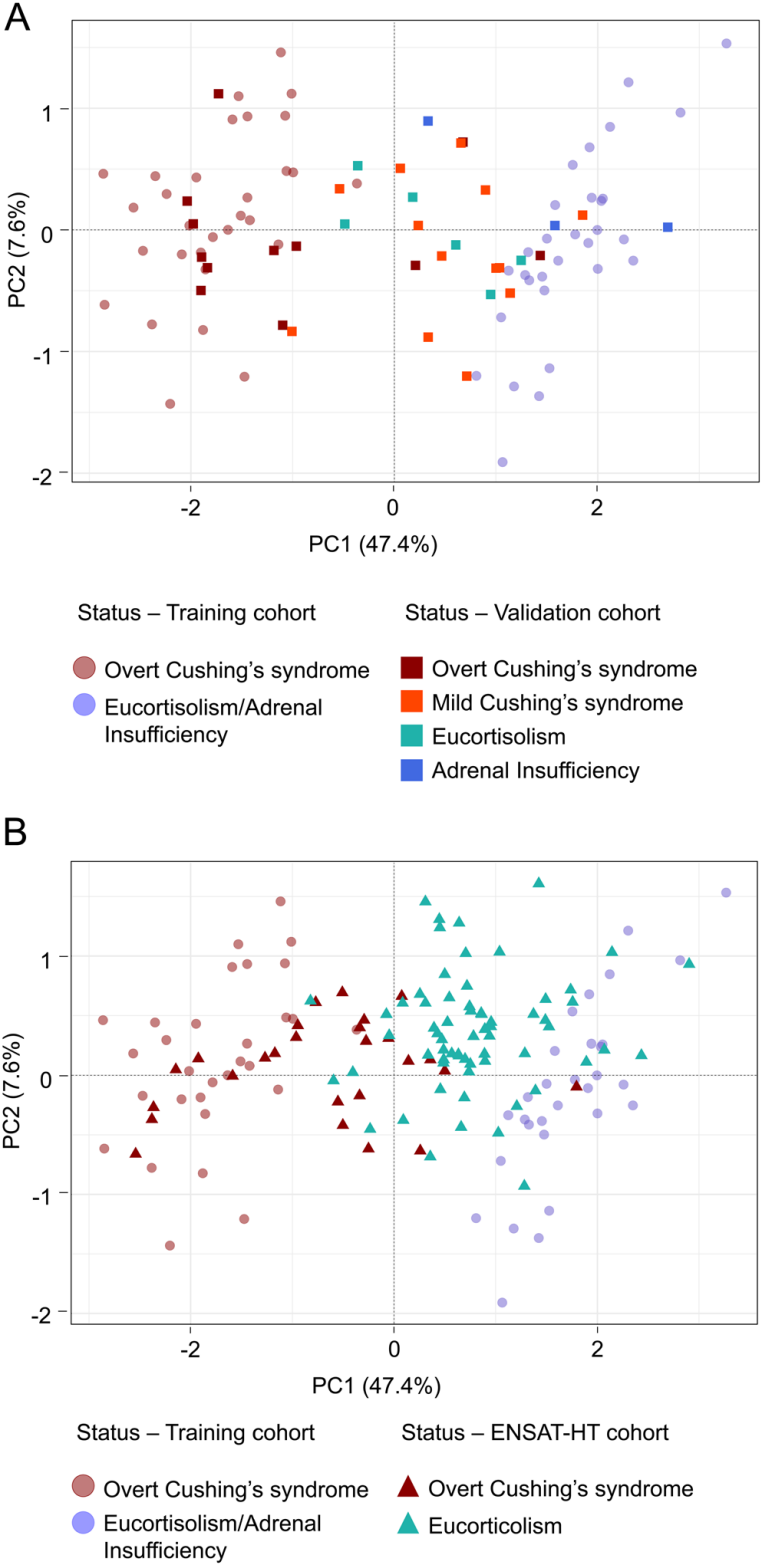
Discrimination of samples based on the 29-CpGs methylation predictor. (A) Samples projection based on the two principle components (PC1, PC2) of unsupervised PCA performed using the 29-CpG sites selected by Lasso regression on the training cohort. In faint circles are presented the samples from the training cohort, on which the optimization of CpG selection was operated. In bright squares are presented the samples from the validation cohort. (B) Similar projection using a second independent validation cohort, with samples from the ENSAT-HT cohort presented in bright triangles.

To select a limited set of CpG sites predicting the glucocorticoid status, we performed a Lasso-penalized linear regression on the training cohort, starting from the 52 727 most variable CpG sites. Twenty-nine CpG sites were selected (Supplementary Table 9), properly discriminating overt Cushing’s syndrome in the training cohort, with confirmation in the validation cohort (Fig. 5A). A 29-CpGs methylation predictor was generated by combining the M-values of the 29 selected CpG sites weighted by their Lasso coefficients. This predictor was significantly associated with the glucocorticoid status in the validation cohort (odds ratio: 1.58; 95% CI: 1.25–2.08; *P* -value < 0.001).

The 29-CpGs methylation predictor was then tested on the ENSAT-HT cohort, a second independent validation cohort (26 overt Cushing’s syndrome and 65 eucortisolism samples). Samples were properly classified as Cushing’s syndrome and eucortisolism respectively (Fig. 5B) and the prediction value could be confirmed (odds ratio: 1.10; 95% CI: 1.07–1.12; *P* -value < 0.001), corresponding to an accuracy of 0.84.

One of the 29 selected CpG sites was located in the *FKBP5* gene locus. Among the CpG sites in this locus, the one with the highest correlation to the 29-CpGs methylation predictor (Pearson’s r = −0.89) (Supplementary Table 10) properly discriminated on its own overt Cushing’s syndrome from other samples (Supplementary Fig. 4).

We finally tested to which extent the glucocorticoid effect on blood methylome was related to glucocorticoid-induced white blood cell count variations. In a multivariate model combining the 29-CpGs methylation predictor and the neutrophils proportion, the 29-CpGs methylation predictor remained significant (logistic regression *P* -value < 0.001; Table 2).

**Table 2:** Multivariate model combining methylome and neutrophils predictors on glucocorticoid status. Two statuses were considered: Cushing’s syndrome (overt or mild) and no Cushing’s syndrome (eucortisolism or adrenal insufficiency).

Variables	OR	95% CI	*P*
29-CpGs methylation predictor	2.02	1.51–3.0	<0.001
Proportion of neutrophils (%)	0.97	0.87–1.08	0.6

### Candidate CpG sites predicting Cushing’s syndrome-related complications

**Figure 6:**
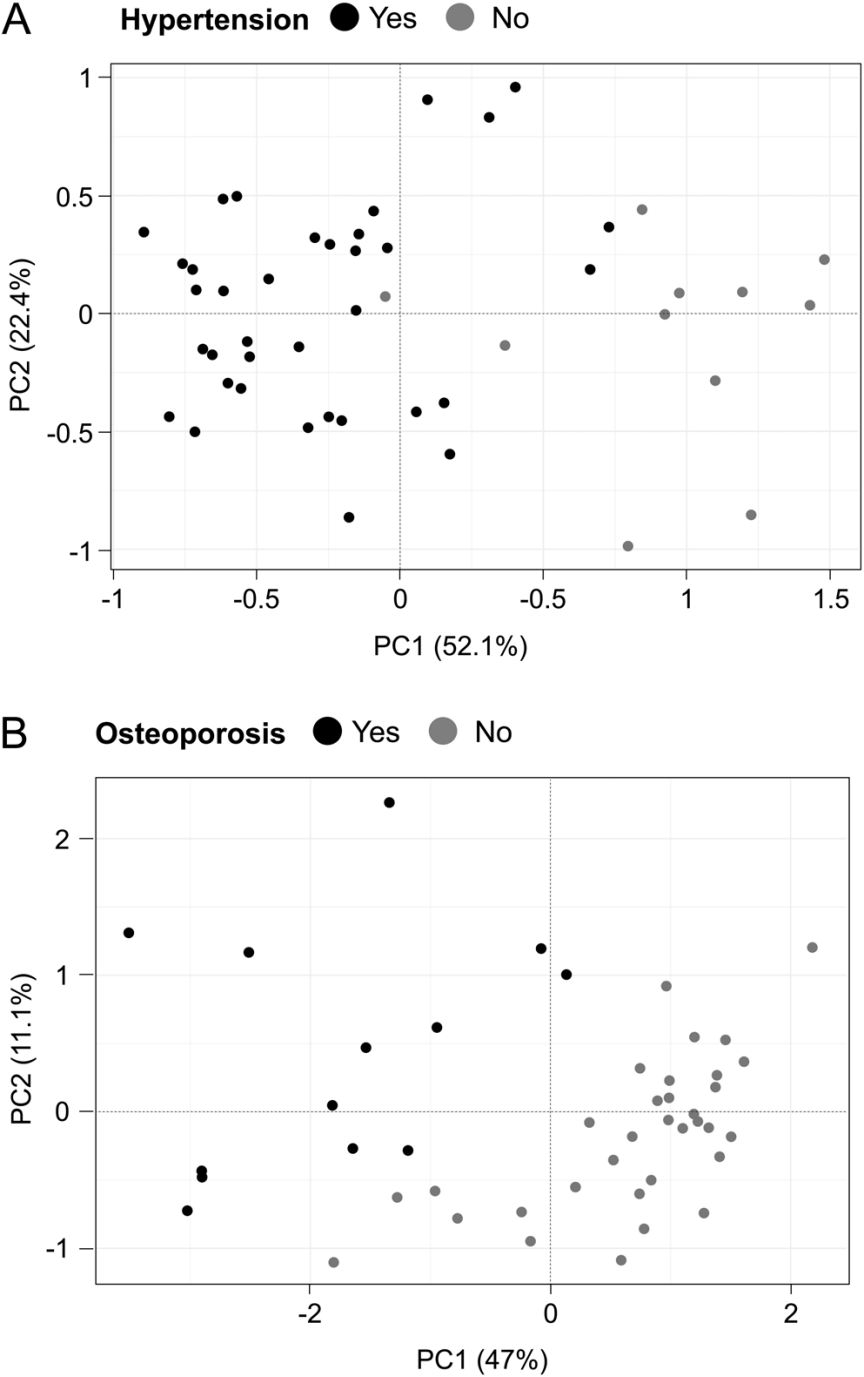
Discrimination of glucocorticoid-related complications. Projection of the 47 Cushing’s syndrome samples based on the 2 principle components (PC1, PC2) of unsupervized PCA performed using the CpG sites selected by Lasso regression discriminating hypertension (4 CpGs, panel A) and osteoporosis (14 CpGs, panel B).

In order to address whether blood DNA methylation is associated with specific glucocorticoid-related complications, we performed an exploratory Lasso regression analysis on Cushing’s syndrome samples from the 47 patients. A combination of four CpG sites was able to discriminate Cushing’s syndrome patients with and without hypertension. Similarly, 14 CpG sites discriminated patients with and without osteoporosis (Fig. 6 and Supplementary Tables 11, 12). The combination of the methylation level of selected CpG sites for hypertension and osteoporosis was not correlated with 24-h urine-free cortisol (Pearson’s r = 0.06 and r = −0.05 for hypertension and osteoporosis, respectively). No combination of CpG sites was able to discriminate Cushing’s syndrome patients with and without diabetes.

## Discussion

In this study, we demonstrated that whole blood methylome quantified biological glucocorticoid action. This biomarker was able to discriminate glucocorticoid excess from eucortisolism and adrenal insufficiency, independently from hormone assays. This new insight may contribute to overcome common pitfalls in Cushing’s syndrome diagnosis and management (35, 36). In clinical practice, such a tool would be of limited benefit in case of overt Cushing’s syndrome, when clinical signs and hormone assays straightforwardly establish the diagnosis. However, a non-hormonal biomarker, directly measuring glucocorticoid action, could particularly help in three conditions: (i) in patients with mild autonomous cortisol secretion, to decide between surveillance and surgical correction of glucocorticoid excess; (ii) in patients under local or low-dose systemic glucocorticoid treatments, to assess the global glucocorticoid level. Indeed, morning plasma cortisol is often low in these patients, and cannot properly assess the glucocorticoid level; this low glucocorticoid level may either reflect mild glucocorticoid excess with negative feedback on endogenous cortisol production, or adrenal insufficiency resulting from prolonged adrenal blockade (37); and (iii) in patients with adrenal insufficiency, to determine the optimal glucocorticoid supplementation (38). At this stage, though, the performance of our biomarker in these intermediate conditions and in exogenous Cushing’s syndrome remains to be established, as well as its clinical relevance.

Here, we have analysed the global methylation state of blood DNA. Blood DNA is easy to obtain and DNA methylation marks are robust and convenient to investigate. In addition, DNA methylation is highly variable, enabling its use as a suitable biomarker.

Our analysis revealed a global DNA hypomethylation signature associated with endogenous Cushing’s syndrome, demonstrating the direct impact of glucocorticoids on DNA methylation. DNA hypomethylation was already observed in an experimental model of mice treated with exogenous glucocorticoids (16). We could delineate a methylation gradient reflecting the relative degree of glucocorticoid excess, ranging from overt Cushing’s syndrome to mild Cushing’s syndrome/eucortisolism, and adrenal insufficiency. Of note, this signature derives from whole blood, an admixture of various cell types with potentially cell-dependent methylation patterns. Indeed, glucocorticoids have a direct effect on white blood cell count, inducing neutrophils increase and lymphocytes decrease (33, 34). However, we could precisely infer white blood cell count from methylome profiles for each sample, and demonstrate that methylome prediction of glucocorticoid status remained significant after adjustment on white blood cell composition and therefore that methylome profiles variations do not only reflect blood composition variations. In addition, the methylome signature provided here in terms of differentially methylated CpG sites and regions is adjusted for white blood cell composition, thus focusing on differences not related to white blood cell composition. This suggests a global impact of glucocorticoids on methylation of some DNA regions, irrespective of their tissue of origin.

After correction of glucocorticoid excess, long-term consequences have been reported (39, 40, 41, 42). An easily measurable biomarker reflecting the dynamic of biological changes in time, such as blood DNA methylation, could help monitoring patients during follow-up. We observed that blood DNA hypomethylation progressively recovers in the years following remission. Similarly, a subtle DNA hypomethylation was observed by Glad *et al*. several years after Cushing’s syndrome correction (43). The authors could correlate some blood methylation levels at specific genomic regions with long-term Cushing-associated neuropsychological sequels. Whether blood DNA methylation can properly help to monitor recovery after Cushing’s syndrome correction remains to be specifically explored.

In case of eucortisolism and mild Cushing’s syndrome, our methylation predictor showed intermediate classification, between overt Cushing’s syndrome and adrenal insufficiency, sometimes discrepant from clinical appraisal. In such conditions, it was not possible to assess whether the methylation predictor was more accurate than clinical evaluation. In such cases, the most relevant judgmental criteria would be the correlation with long-term complications. Therefore, we explored the association between methylation and some well-appreciated glucocorticoid-related complications. Some selected CpG sites were identified as associated with hypertension and osteoporosis. New cohorts would be necessary to validate this association. In addition, whether these markers are specific to glucocorticoid excess remains to be established in prospective trials. Indeed, the inclusion of patients with no glucocorticoid excess would serve as negative controls, necessary for discarding markers of hypertension, diabetes, or osteoporosis not related to glucocorticoid excess.

Whole genome methylation profiling is not easily achievable in clinical routine, thus representing a limitation in using this new marker. A technology transfer to targeted methylation assays would be required, such as pyrosequencing, methylation specific-MLPA, or methylation-specific high-resolution melting analysis (44). An alternative would be the identification of surrogate DNA regions recapitulating this global information. One region could be represented by the *FKBP5* gene locus, whose methylation and expression have been demonstrated to be modulated by glucocorticoids in different tissues (45, 46, 47, 48, 49, 50), with hypomethylation of the promoter region associated with increased gene expression. In this study, we also identified the *FKBP5* promoter region methylation as strongly associated with glucocorticoid excess and negatively correlated with *FKBP5* gene expression. Particularly, one single CpG site from the *FKBP5* gene locus could discriminate overt Cushing’s syndrome samples.

Sample size is another limitation of this study, with 94 samples from 47 patients. However, samplings were performed at different times of the disease, corresponding to different glucocorticoid statuses. Additional samples from the ENSAT-HT cohort allowed to further validate the performance of our marker. Further extending the cohort would help to confirm this finding, helping to better characterize the association with specific complications of glucocorticoid excess.

In conclusion, glucocorticoids induce a dynamic whole blood DNA methylation signature. This signature could be used as a biomarker for assessing glucocorticoid action independently from hormone assays.

## Supplementary Material

Supplementary Materials

Supplementary Table 1

Supplementary Table 2

Supplementary Table 3

Supplementary Table 4

Supplementary Table 5 – Gene set enrichment analysis: overt Cushing’s syndrome versus eucortisolism

Supplementary Table 6 – Gene set enrichment analysis: overt Cushing’s syndrome versus adrenal insufficiency

Supplementary Table 7

Supplementary Table 8

Supplementary Table 9 - 29-Lasso selected CpG sites

Supplementary Table 10

Supplementary Table 11 – Hypertension-associated CpG sites

Supplementary Table 12 – Osteoporosis-associated CpG sites

Supplementary Figure 1

Supplementary Figure 2

Supplementary Figure 3

Supplementary Figure 4

## Declaration of interest

Guillaume Assié is on the editorial board of EJE. Guillaume Assié was not involved in the review or editorial process for this paper, on which he is listed as an author.

## Funding

This project has received funding from the European Union’s Horizon 2020 research and innovation programme under grant agreement No633983, the Programme Hospitalier de Recherche Clinique ‘CompliCushing’ (PHRC AOM 12-002-0064), Agence Nationale pour la Recherche (ANR-18-CE14-0008-01), the Else Kröner-Fresenius Stiftung (2012_A103 and 2015_A228 to M R) and the Deutsche Forschungsgemeinschaft (DFG) within the CRC/Transregio 205/1 ‘The Adrenal: Central Relay in Health and Disease’ (to M R, F B, A R).

## Data access

Methylome data generated in this study have been deposited in the ArrayExpress database at EMBL-EBI (http://www.ebi.ac.uk/arrayexpress/experiments/E-MTAB-10092).

## Author contribution statement

R A, G A: conceptualization; L B, L B, A R, F B S, A B, L A, C S, F C, G P R, T A W, C K L, F B, M R, J B, G A: clinical data and sample collection; M C Z, M R, F B, J B, G A: project administration and ethical aspects management; R A, C G, K P: samples handling and genomic data generation; R A, A J, A S, T L, M N, S A, G A: bioinformatics and statistical analyses; R A, G A: original draft preparation. All authors: manuscript review and editing. F B, M R, J B and G A: these authors equally contributed.

## References

[1] Laugesen K Jørgensen JOL Petersen I Sørensen HT. Fifteen-year nationwide trends in systemic glucocorticoid drug use in Denmark. European Journal of Endocrinology 2019 181 267–273. (10.1530/EJE-19-0305) 31269470

[2] Ekström M Nwaru BI Hasvold P Wiklund F Telg G Janson C. Oral corticosteroid use, morbidity and mortality in asthma: a nationwide prospective cohort study in Sweden. Allergy 2019 74 2181–2190. (10.1111/all.13874) 31095758 PMC6899917

[3] Wengander S Trimpou P Papakokkinou E Ragnarsson O. The incidence of endogenous Cushing’s syndrome in the modern era. Clinical Endocrinology 2019 91 263–270. (10.1111/cen.14014) 31094003

[4] Fassnacht M Arlt W Bancos I Dralle H Newell-Price J Sahdev A Tabarin A Terzolo M Tsagarakis S Dekkers OM. Management of adrenal incidentalomas: European Society of Endocrinology Clinical Practice Guideline in collaboration with the European Network for the study of adrenal tumors. European Journal of Endocrinology 2016 175 G1–G34. (10.1530/EJE-16-0467) 27390021

[5] Javanmard P Duan D Geer EB. Mortality in patients with endogenous Cushing’s syndrome. Endocrinology and Metabolism Clinics of North America 2018 47 313–333. (10.1016/j.ecl.2018.02.005) 29754634

[6] Pivonello R Isidori AM De Martino MC Newell-Price J Biller BMK Colao A. Complications of Cushing’s syndrome: state of the art. Lancet. Diabetes and Endocrinology 2016 4 611–629. (10.1016/S2213-8587(1600086-3) 27177728

[7] Valassi E Santos A Yaneva M Tóth M Strasburger CJ Chanson P Wass JAH Chabre O Pfeifer M Feelders RA The European Registry on Cushing’s syndrome: 2-year experience. Baseline demographic and clinical characteristics. European Journal of Endocrinology 2011 165 383–392. (10.1530/EJE-11-0272) 21715416

[8] Hahner S Spinnler C Fassnacht M Burger-Stritt S Lang K Milovanovic D Beuschlein F Willenberg HS Quinkler M Allolio B. High incidence of adrenal crisis in educated patients with chronic adrenal insufficiency: a prospective study. Journal of Clinical Endocrinology and Metabolism 2015 100 407–416. (10.1210/jc.2014-3191) 25419882

[9] Di Dalmazi G Vicennati V Garelli S Casadio E Rinaldi E Giampalma E Mosconi C Golfieri R Paccapelo A Pagotto U Cardiovascular events and mortality in patients with adrenal incidentalomas that are either non-secreting or associated with intermediate phenotype or subclinical Cushing’s syndrome: a 15-year retrospective study. Lancet. Diabetes and Endocrinology 2014 2 396–405. (10.1016/S2213-8587(1370211-0) 24795253

[10] Petramala L Olmati F Concistrè A Russo R Mezzadri M Soldini M De Vincentis G Iannucci G De Toma G Letizia C. Cardiovascular and metabolic risk factors in patients with subclinical Cushing. Endocrine 2020 70 150–163. (10.1007/s12020-020-02297-2) 32300953

[11] Schübeler D Function and information content of DNA methylation. Nature 2015 517 321–326. (10.1038/nature14192) 25592537

[12] Logue MW Miller MW Wolf EJ Huber BR Morrison FG Zhou Z Zheng Y Smith AK Daskalakis NP Ratanatharathorn A An epigenome-wide association study of posttraumatic stress disorder in US veterans implicates several new DNA methylation loci. Clinical Epigenetics 2020 12 46. (10.1186/s13148-020-0820-0) 32171335 PMC7071645

[13] Martin C Cho YE Kim H Yun S Kanefsky R Lee H Mysliwiec V Cashion A Gill J. Altered DNA methylation patterns associated with clinically relevant increases in PTSD symptoms and PTSD symptom profiles in military personnel. Biological Research for Nursing 2018 20 352–358. (10.1177/1099800418758951) 29514460 PMC5993080

[14] Rutten BPF Vermetten E Vinkers CH Ursini G Daskalakis NP Pishva E de Nijs L Houtepen LC Eijssen L Jaffe AE Longitudinal analyses of the DNA methylome in deployed military servicemen identify susceptibility loci for post-traumatic stress disorder. Molecular Psychiatry 2018 23 1145–1156. (10.1038/mp.2017.120) 28630453 PMC5984086

[15] Vinkers CH Geuze E van Rooij SJH Kennis M Schür RR Nispeling DM Smith AK Nievergelt CM Uddin M Rutten BPF Successful treatment of post-traumatic stress disorder reverses DNA methylation marks. Molecular Psychiatry 2021 26 1264–1271. (10.1038/s41380-019-0549-3) 31645664

[16] Seifuddin F Wand G Cox O Pirooznia M Moody L Yang X Tai J Boersma G Tamashiro K Zandi P Genome-wide methyl-Seq analysis of blood-brain targets of glucocorticoid exposure. Epigenetics 2017 12 637–652. (10.1080/15592294.2017.1334025) 28557603 PMC5687336

[17] Argentieri MA Nagarajan S Seddighzadeh B Baccarelli AA Shields AE. Epigenetic pathways in human disease: the impact of DNA methylation on stress-related pathogenesis and current challenges in biomarker development. EBiomedicine 2017 18 327–350. (10.1016/j.ebiom.2017.03.044) 28434943 PMC5405197

[18] Fries GR Gassen NC Rein T. The FKBP51 glucocorticoid receptor co-chaperone: regulation, function, and implications in health and disease. International Journal of Molecular Sciences 2017 18 2614. (10.3390/ijms18122614) PMC5751217 29206196

[19] Bancos I Hatipoglu BA Yuen KCJ Chandramohan L Chaudhari S Moraitis AG. Evaluation of FKBP5 as a cortisol activity biomarker in patients with ACTH-dependent Cushing syndrome. Journal of Clinical and Translational Endocrinology 2021 24 100256. (10.1016/j.jcte.2021.100256) 34258233 PMC8260880

[20] Nieman LK Biller BMK Findling JW Newell-Price J Savage MO Stewart PM Montori VM. The diagnosis of Cushing’s syndrome: an Endocrine Society Clinical Practice Guideline. Journal of Clinical Endocrinology and Metabolism 2008 93 1526–1540. (10.1210/jc.2008-0125) 18334580 PMC2386281

[21] Aryee MJ Jaffe AE Corrada-Bravo H Ladd-Acosta C Feinberg AP Hansen KD Irizarry RA. Minfi: a flexible and comprehensive bioconductor package for the analysis of Infinium DNA methylation microarrays. Bioinformatics 2014 30 1363–1369. (10.1093/bioinformatics/btu049) 24478339 PMC4016708

[22] Touleimat N Tost J. Complete pipeline for Infinium(®) human methylation 450K BeadChip data processing using subset quantile normalization for accurate DNA methylation estimation. Epigenomics 2012 4 325–341. (10.2217/epi.12.21) 22690668

[23] Tian Y Morris TJ Webster AP Yang Z Beck S Feber A Teschendorff AE. ChAMP: updated methylation analysis pipeline for Illumina BeadChips. Bioinformatics 2017 33 3982–3984. (10.1093/bioinformatics/btx513) 28961746 PMC5860089

[24] Teschendorff AE Menon U Gentry-Maharaj A Ramus SJ Gayther SA Apostolidou S Jones A Lechner M Beck S Jacobs IJ An epigenetic signature in peripheral blood predicts active ovarian cancer. PLoS ONE 2009 4 e8274. (10.1371/journal.pone.0008274) PMC2793425 20019873

[25] Johnson WE Li C Rabinovic A. Adjusting batch effects in microarray expression data using empirical Bayes methods. Biostatistics 2007 8 118–127. (10.1093/biostatistics/kxj037) 16632515

[26] Houseman EA Accomando WP Koestler DC Christensen BC Marsit CJ Nelson HH Wiencke JK Kelsey KT. DNA methylation arrays as surrogate measures of cell mixture distribution. BMC Bioinformatics 2012 13 86. (10.1186/1471-2105-13-86) PMC3532182 22568884

[27] Du P Kibbe WA Lin SM. lumi: a pipeline for processing Illumina microarray. Bioinformatics 2008 24 1547–1548. (10.1093/bioinformatics/btn224) 18467348

[28] Ritchie ME Phipson B Wu D Hu Y Law CW Shi W Smyth GK. limma powers differential expression analyses for RNA-sequencing and microarray studies. Nucleic Acids Research 2015 43 e47. (10.1093/nar/gkv007) PMC4402510 25605792

[29] Phipson B Maksimovic J Oshlack A. missMethyl: an R package for analyzing data from Illumina’s HumanMethylation450 platform. Bioinformatics 2016 32 286–288. (10.1093/bioinformatics/btv560) 26424855

[30] Geeleher P Hartnett L Egan LJ Golden A Raja Ali RA Seoighe C. Gene-set analysis is severely biased when applied to genome-wide methylation data. Bioinformatics 2013 29 1851–1857. (10.1093/bioinformatics/btt311) 23732277

[31] Peters TJ Buckley MJ Statham AL Pidsley R Samaras K V Lord R Clark SJ Molloy PL. De novo identification of differentially methylated regions in the human genome. Epigenetics and Chromatin 2015 8 6. (10.1186/1756-8935-8-6) PMC4429355 25972926

[32] Friedman J Hastie T Tibshirani R. Regularization paths for generalized linear models via coordinate descent. Journal of Statistical Software 2010 33 1–22. (10.18637/jss.v033.i01) 20808728 PMC2929880

[33] Masri-Iraqi H Robenshtok E Tzvetov G Manistersky Y Shimon I. Elevated white blood cell counts in Cushing’s disease: association with hypercortisolism. Pituitary 2014 17 436–440. (10.1007/s11102-013-0522-0) 24078318

[34] Nakagawa M Terashima T D’yachkova Y Bondy GP Hogg JC van Eeden SF. Glucocorticoid-induced granulocytosis: contribution of marrow release and demargination of intravascular granulocytes. Circulation 1998 98 2307–2313. (10.1161/01.cir.98.21.2307) 9826319

[35] Bansal V Asmar NE Selman WR Arafah BM. Pitfalls in the diagnosis and management of Cushing’s syndrome. Neurosurgical Focus 2015 38 E4. (10.3171/2014.11.FOCUS14704) 25639322

[36] Nieman LK Diagnosis of Cushing’s syndrome in the modern era. Endocrinology and Metabolism Clinics of North America 2018 47 259–273. (10.1016/j.ecl.2018.02.001) 29754631

[37] Paragliola RM Papi G Pontecorvi A Corsello SM. Treatment with synthetic glucocorticoids and the hypothalamus-pituitary-adrenal axis. International Journal of Molecular Sciences 2017 18 2201. (10.3390/ijms18102201) PMC5666882 29053578

[38] Hahner S Acute adrenal crisis and mortality in adrenal insufficiency: still a concern in 2018! Annales d’Endocrinologie 2018 79 164–166. (10.1016/j.ando.2018.04.015) 29716733

[39] Colao A Pivonello R Spiezia S Faggiano A Ferone D Filippella M Marzullo P Cerbone G Siciliani M Lombardi G. Persistence of increased cardiovascular risk in patients with Cushing’s disease after five years of successful cure. Journal of Clinical Endocrinology and Metabolism 1999 84 2664–2672. (10.1210/jcem.84.8.5896) 10443657

[40] Espinosa-de-Los-Monteros AL Sosa E Martinez N Mercado M. Persistence of Cushing’s disease symptoms and comorbidities after surgical cure: a long-term, integral evaluation. Endocrine Practice 2013 19 252–258. (10.4158/EP12247.OR) 23543030

[41] Lambert JK Goldberg L Fayngold S Kostadinov J Post KD Geer EB. Predictors of mortality and long-term outcomes in treated Cushing’s disease: a study of 346 patients. Journal of Clinical Endocrinology and Metabolism 2013 98 1022–1030. (10.1210/jc.2012-2893) 23393167 PMC3590483

[42] Vermalle M Alessandrini M Graillon T Paladino NC Baumstarck K Sebag F Dufour H Brue T Castinetti F. Lack of functional remission in Cushing’s syndrome. Endocrine 2018 61 518–525. (10.1007/s12020-018-1664-7) 30019306

[43] Glad CAM Andersson-Assarsson JC Berglund P Bergthorsdottir R Ragnarsson O Johannsson G. Reduced DNA methylation and psychopathology following endogenous hypercortisolism – a genome-wide study. Scientific Reports 2017 7 44445. (10.1038/srep44445) PMC5353706 28300138

[44] García-Giménez JL Seco-Cervera M Tollefsbol TO Romá-Mateo C Peiró-Chova L Lapunzina P Pallardó FV. Epigenetic biomarkers: current strategies and future challenges for their use in the clinical laboratory. Critical Reviews in Clinical Laboratory Sciences 2017 54 529–550. (10.1080/10408363.2017.1410520) 29226748 PMC6733278

[45] Lee RS Tamashiro KLK Yang X Purcell RH Huo Y Rongione M Potash JB Wand GS. A measure of glucocorticoid load provided by DNA methylation of Fkbp5 in mice. Psychopharmacology 2011 218 303–312. (10.1007/s00213-011-2307-3) 21509501 PMC3918452

[46] Resmini E Santos A Aulinas A Webb SM Vives-Gilabert Y Cox O Wand G Lee RS. Reduced DNA methylation of FKBP5 in Cushing’s syndrome. Endocrine 2016 54 768–777. (10.1007/s12020-016-1083-6) 27664120 PMC6391874

[47] Wiechmann T Röh S Sauer S Czamara D Arloth J Ködel M Beintner M Knop L Menke A Binder EB Identification of dynamic glucocorticoid-induced methylation changes at the FKBP5 locus. Clinical Epigenetics 2019 11 83. (10.1186/s13148-019-0682-5) PMC6533766 31122292

[48] Winkler BK Lehnert H Oster H Kirchner H Harbeck B. FKBP5 methylation as a possible marker for cortisol state and transient cortisol exposure in healthy human subjects. Epigenomics 2017 9 1279–1286. (10.2217/epi-2017-0057) 28875708

[49] Cox OH Song HY Garrison-Desany HM Gadiwalla N Carey JL Menzies J Lee RS. Characterization of glucocorticoid-induced loss of DNA methylation of the stress-response gene *Fkbp5* in neuronal cells. Epigenetics 2020 16 1377–1397. (10.1080/15592294.2020.1864169) PMC8813076 33319620

[50] Chatzittofis A Boström ADE Ciuculete DM Öberg KG Arver S Schiöth HB Jokinen J. HPA axis dysregulation is associated with differential methylation of CpG-sites in related genes. Scientific Reports 2021 11 20134. (10.1038/s41598-021-99714-x) 34635736 PMC8505644

