# Supplementary material for: Identification of glucocorticoid-related molecular signature by whole blood methylome analysis
Source: Eur J Endocrinol. 2021 Dec 16;186(2):297–308. doi: 10.1530/EJE-21-0907 (PMC8789024; doi:10.1530/EJE-21-0907)
Supplement: Supplementary Materials [file supplementary_material.pdf]

Supplementary Methods

RNA extraction and RT-qPCR

Total RNA was extracted from whole blood samples, collected into PAXgene tubes (PreAnalytiX, Hombrechtikon, Switzerland), by using a specific extraction kit (Qiagen, Hilden, Germany). The expression levels of target genes were determined by means of real-time PCR using a LightCycler Fast Start SYBR Green kit (Roche, Basil, Switzerland) according to the manufacturer's instructions. Relative quantification of target cDNA was determined by calculating the difference in cross-threshold (CT) values after normalization to *PPIA* (Cyclophilin A) signals (ΔΔCT method). Primer sequences for target genes were the following: for *FKBP5*, 5'AAGAGTGGGGAATGGTGAGG (Forward primer) and 5'ATGGTAGCCACCCCAATGTC (Reverse primer); for *PPIA*, 5'ATGGCACTGGTGGCAAGTCC (Forward primer) and 5'TTGCCATTCCTGGACCCAAA (Reverse primer). Amplification was performed at 60°C.

Supplementary Tables

Supplementary Table 1 – Samples characteristics

“Supplementary_Table1.xlsx” file

Supplementary Table 2 – ENSAT-HT independent cohort samples

“Supplementary_Table2.xlsx” file

Supplementary Table 3 – Proportion of measured and estimated neutrophils and leukocytes

“Supplementary_Table3.xlsx” file

Supplementary Table 4 – Significant differentially methylated CpG sites in overt Cushing’s syndrome

“Supplementary_Table4.xlsx” file

Supplementary Table 5 – Gene set enrichment analysis: overt Cushing’s syndrome versus eucortisolism

“Supplementary_Table5.docx” file

Supplementary Table 6 – Gene set enrichment analysis: overt Cushing’s syndrome versus adrenal insufficiency

“Supplementary_Table6.docx” file

Supplementary Table 7 – Differentially methylated regions: overt Cushing’s syndrome versus eucortisolism

“Supplementary_Table7.xlsx” file

Supplementary Table 8 – Differentially methylated regions: overt Cushing's syndrome versus adrenal insufficiency

"Supplementary_Table8.xlsx" file

Supplementary Table 9 - 29-Lasso selected CpG sites

"Supplementary_Table9.docx" file

Supplementary Table 10 – *FKBP5* gene locus-associated CpG sites

"Supplementary_Table10.xlsx" file

Supplementary Table 11 – Hypertension-associated CpG sites

"Supplementary_Table11.docx" file

Supplementary Table 12 – Osteoporosis-associated CpG sites

"Supplementary_Table12.docx" file

Supplementary Figures

Supplementary Figure 1. Correlation between measured and estimated neutrophils (A) and lymphocytes (B).

Supplementary Figure 2. Components of variation in the whole methylome dataset. A) Scree plot representing the percentage of explained variability by the first five principal components of PCA performed on the whole dataset (n=731,635 CpG sites, n=94 samples). PC-1 accounts for most of the variability. B) Singular value decomposition (SVD) plot assessing the correlation between the first five significant components of variation in the dataset and biological factors of interest (Status –overt Cushing's syndrome, mild Cushing's syndrome, eucortisolism, adrenal insufficiency-, age, sex, proportion of neutrophils).

Supplementary Figure 3. Distribution of differentially methylated CpG sites (overt Cushing's syndrome vs. adrenal insufficiency: n=7120). A) Distribution relative to genome CpG enrichment. B) Distribution relative to gene locus structure. C) Genomic distribution. Highlighted in black, the CpG sites located in the *FKBP5* gene locus on chromosome 6.

Supplementary Figure 4. Methylation level of one single CpG site from the *FKBP5* gene locus in Cushing's syndrome samples. A) Boxplot representation of cg19226017 methylation level, a CpG site belonging to the *FKBP5* promoter region and showing the highest correlation with the 29-CpG methylation predictor. B) Boxplot representation of the methylation level of the same *FKBP5*-associated CpG site -cg19226017- in the ENSAT-HT cohort. ***p-value$<10^{-5}$.
