# Supplementary material for: Identification of glucocorticoid-related molecular signature by whole blood methylome analysis
Source: Eur J Endocrinol. 2021 Dec 16;186(2):297–308. doi: 10.1530/EJE-21-0907 (PMC8789024; doi:10.1530/EJE-21-0907)
Supplement: Supplementary Table 5 – Gene set enrichment analysis: overt Cushing’s syndrome versus eucortisolism [file supplementary_table_5.pdf]

Supplementary Table 5 – Gene set enrichment analysis: overt Cushing's syndrome versus eucortisolism

	**ONTOLOGY**	**TERM**	**N**	**DE**	**P.DE**	**FDR**
GO:0043312	BP	neutrophil degranulation	481	48	2.53621e-09	4.38765e-05
GO:0070062	CC	extracellular exosome	2162	122.5	0.000188972	0.959715
GO:0035425	BP	autocrine signaling	7	3	0.000255442	0.959715
GO:0032691	BP	negative regulation of interleukin-1 beta production	15	4	0.000311296	0.959715
GO:0050853	BP	B cell receptor signaling pathway	28	8	0.000338579	0.959715
GO:0046627	BP	negative regulation of insulin receptor signaling pathway	29	7	0.000414997	0.959715
GO:0005829	CC	cytosol	4871	262	0.000435416	0.959715
GO:0004697	MF	protein kinase C activity	15	6	0.000443799	0.959715
GO:1904813	CC	ficolin-1-rich granule lumen	124	14.5	0.000529842	0.967862
GO:0030316	BP	osteoclast differentiation	30	7	0.000559458	0.967862

Top 10 enriched pathways. BP = Biological process; CC = Cellular Component; MF = Molecular Function
