# Supplementary material for: Identification of glucocorticoid-related molecular signature by whole blood methylome analysis
Source: Eur J Endocrinol. 2021 Dec 16;186(2):297–308. doi: 10.1530/EJE-21-0907 (PMC8789024; doi:10.1530/EJE-21-0907)
Supplement: Supplementary Table 6 – Gene set enrichment analysis: overt Cushing’s syndrome versus adrenal insufficiency [file supplementary_table_6.pdf]

Supplementary Table 6 – Gene set enrichment analysis: overt Cushing's syndrome versus adrenal insufficiency

	ONTOLOGY	TERM	N	DE	P.DE	FDR
GO:0043312	BP	neutrophil degranulation	481	154.8	3.10037e-19	5.36364e-15
GO:0002250	BP	adaptive immune response	279	47	2.0963e-07	0.0018133
GO:0006954	BP	inflammatory response	288	77.3	3.92353e-07	0.00226257
GO:0005764	CC	lysosome	230	70	9.18582e-07	0.00391109
GO:0050853	BP	B cell receptor signaling pathway	28	19	1.13037e-06	0.00391109
GO:0042101	CC	T cell receptor complex	16	12	3.03243e-06	0.0087435
GO:0035579	CC	specific granule membrane	91	34	3.80736e-06	0.00940963
GO:0042110	BP	T cell activation	42	21	6.45125e-06	0.0139508
GO:0071222	BP	cellular response to lipopolysaccharide	133	41.6	1.40973e-05	0.0270981
GO:0032930	BP	positive regulation of superoxide anion generation	17	11	2.52111e-05	0.0436151

Top 10 enriched pathways. BP = Biological process; CC = Cellular Component
