# Supplementary material for: Identification of glucocorticoid-related molecular signature by whole blood methylome analysis
Source: Eur J Endocrinol. 2021 Dec 16;186(2):297–308. doi: 10.1530/EJE-21-0907 (PMC8789024; doi:10.1530/EJE-21-0907)
Supplement: Supplementary Table 9 - 29-Lasso selected CpG sites [file supplementary_table_9.pdf]

Supplementary Table 9 - 29-Lasso selected CpG sites

CpG name	Chromosome	Genome position (GRCh37)	Islands Name	Relation to Island	Gene Name	Gene Locus	Delta M-value (overt Cushing's syndrome vs eucortisolism/ adrenal insufficiency)	Lasso coefficient
cg03546163	chr6	35654363	chr6:35655607-35656856	N_Shore	FKBP5	5'UTR	-0.9	-0.5
cg12028969	chr11	34393189		OpenSea		IGR	-1.01	-1.01
cg27662789	chr12	43666281		OpenSea		IGR	0.61	0.11
cg02527360	chr11	128194339		OpenSea		IGR	0.69	0.19
cg15013768	chr2	62132624		OpenSea	COMMD1	TSS200	-0.34	-0.51
cg14195992	chr8	48265917		OpenSea	KIAA0146	Body	-1.01	-0.14
cg04906211	chr15	64762285		OpenSea		IGR	0.44	0.04
cg03036592	chr10	101287839	chr10:101287162-101287920	Island		IGR	-0.7	-0.38
cg05224662	chr2	127950633		OpenSea	CYP27C1	Body	0.42	0.78
cg07369212	chr4	169432922		OpenSea	PALLD	Body	0.83	0.15
cg23525061	chr16	50402411		OpenSea	BRD7	Body	0.38	0.1
cg24166814	chr2	56067277		OpenSea		IGR	-0.88	-3.2
cg20916120	chr11	20903027		OpenSea	NELL1	Body	0.34	0.05
cg09546359	chr5	102594340	chr5:102594435-102595550	N_Shore	C5orf30	TSS200	0.46	0.32
cg18642369	chr13	99651231		OpenSea	DOCK9	Body	0.8	0.56
cg12754982	chr6	160409942		OpenSea	IGF2R	Body	-0.86	-0.62
cg22277636	chr2	218621320	chr2:218621279-218621498	Island	DIRC3	TSS200	-0.38	-0.24
cg01617955	chr4	151667406		OpenSea	LRBA	Body	0.41	0.2
cg26277237	chr9	631910		OpenSea	KANK1	5'UTR	0.84	0.49
cg06036471	chr10	93861385		OpenSea	CPEB3	Body	0.8	0.26
cg14101485	chr19	3369759	chr19:3369477-3369913	Island	NFIC	Body	-0.7	-0.77
cg16396907	chr11	114229775		OpenSea		IGR	-0.72	-1.36
cg04871835	chr16	75018965	chr16:75018396-75019245	Island	WDR59	5'UTR	0.34	0.01
cg01666600	chr17	21279561	chr17:21279506-21281574	Island	KCNJ12	TSS200	-0.31	-0.2
cg12864409	chr12	110742270		OpenSea	ATP2A2	Body	-0.71	-0.86
cg12796186	chr1	10458599	chr1:10458548-10459997	Island	PGD	TSS1500	0.31	0.54
cg09859564	chr10	281489		OpenSea	ZMYND11	Body	0.3	1.22
cg18677148	chr17	57712280		OpenSea	CLTC	Body	0.73	0.4
cg26954533	chr19	9546262	chr19:9545783-9546302	Island	ZNF266	TSS200	0.31	0.81
