# Supplementary material for: Identification of glucocorticoid-related molecular signature by whole blood methylome analysis
Source: Eur J Endocrinol. 2021 Dec 16;186(2):297–308. doi: 10.1530/EJE-21-0907 (PMC8789024; doi:10.1530/EJE-21-0907)
Supplement: Supplementary Table 11 – Hypertension-associated CpG sites [file supplementary_table_11.pdf]

Supplementary Table 11 – Hypertension-associated CpG sites

CpG name	Chromosome	Genome position (GRCh37)	Islands Name	Relation to Island	Gene Name	Gene Locus	Lasso coefficient
cg01967073	chr7	48147130		OpenSea	UPP1	Body	-0.12
cg24649335	chr3	14845410		OpenSea			0.07
cg01884612	chr22	31031439	chr22:31031358-31032059	Island	SLC35E4	TSS1500	-0.01
cg03380349	chr16	47492751	chr16:47494697-47495207	N_Shore	ITFG1	5'UTR	-1.14
