# Supplementary material for: Identification of glucocorticoid-related molecular signature by whole blood methylome analysis
Source: Eur J Endocrinol. 2021 Dec 16;186(2):297–308. doi: 10.1530/EJE-21-0907 (PMC8789024; doi:10.1530/EJE-21-0907)
Supplement: Supplementary Table 12 – Osteoporosis-associated CpG sites [file supplementary_table_12.pdf]

Supplementary Table 12 – Osteoporosis-associated CpG sites

CpG name	Chromosome	Genome position (GRCh37)	Islands Name	Relation to Island	Gene Name	Gene Locus	Lasso coefficient
cg16611967	chr16	90144006	chr16:90143683-90144081	Island			0.22
cg00524694	chr5	177387682	chr5:177388753-177389250	N_Shore			-0.05
cg01629329	chr20	2633258	chr20:2632678-2633690	Island	NOP56	1stExon	0.50
cg10563109	chr6	5087749	chr6:5084516-5087032	S_Shore			-0.09
cg04160749	chr8	58172571	chr8:58172076-58173863	Island			0.13
cg12912919	chr7	92079392	chr7:92076699-92077395	S_Shore	GATAD1	Body	0.51
cg09641660	chr1	28198869	chr1:28199031-28199257	N_Shore	THEMIS2	TSS200	0.51
cg19137662	chr20	2633362	chr20:2632678-2633690	Island	NOP56	Body	0.04
cg07789658	chr8	23386185	chr8:23386220-23387386	N_Shore	SLC25A37	TSS200	0.49
cg19174044	chr20	18446362	chr20:18447429-18448341	N_Shore	DZANK1	5'UTR	0.14
cg05880330	chr3	137484517	chr3:137482964-137484454	S_Shore			0.16
cg13557804	chr6	33117976		OpenSea			0.04
cg23603273	chr10	64679766		OpenSea			-0.05
cg19623588	chr2	61149856		OpenSea	REL	3'UTR	-0.24
