# Supplementary figures and images for: Identification of glucocorticoid-related molecular signature by whole blood methylome analysis
Source: Eur J Endocrinol. 2021 Dec 16;186(2):297–308. doi: 10.1530/EJE-21-0907 (PMC8789024; doi:10.1530/EJE-21-0907)

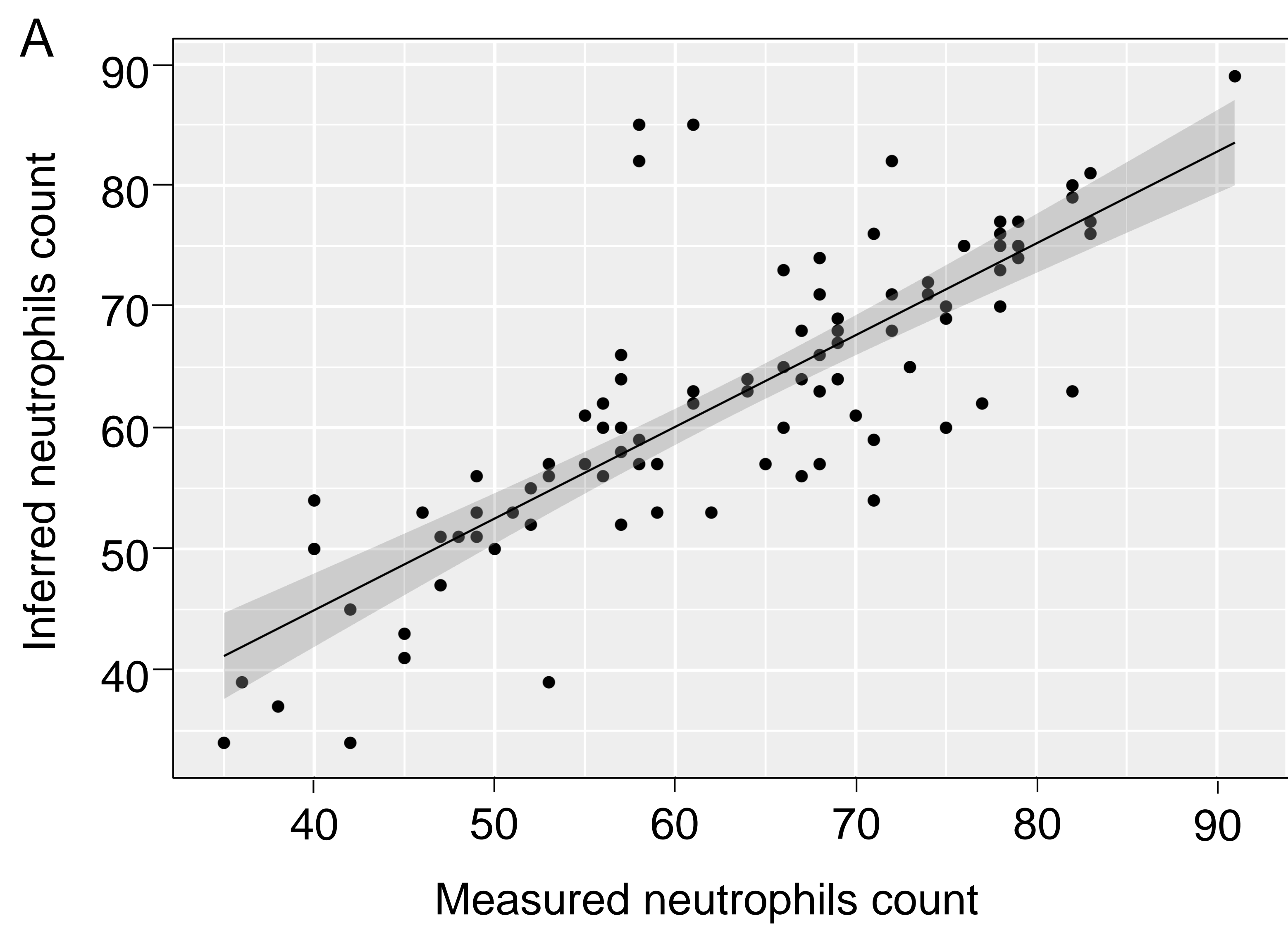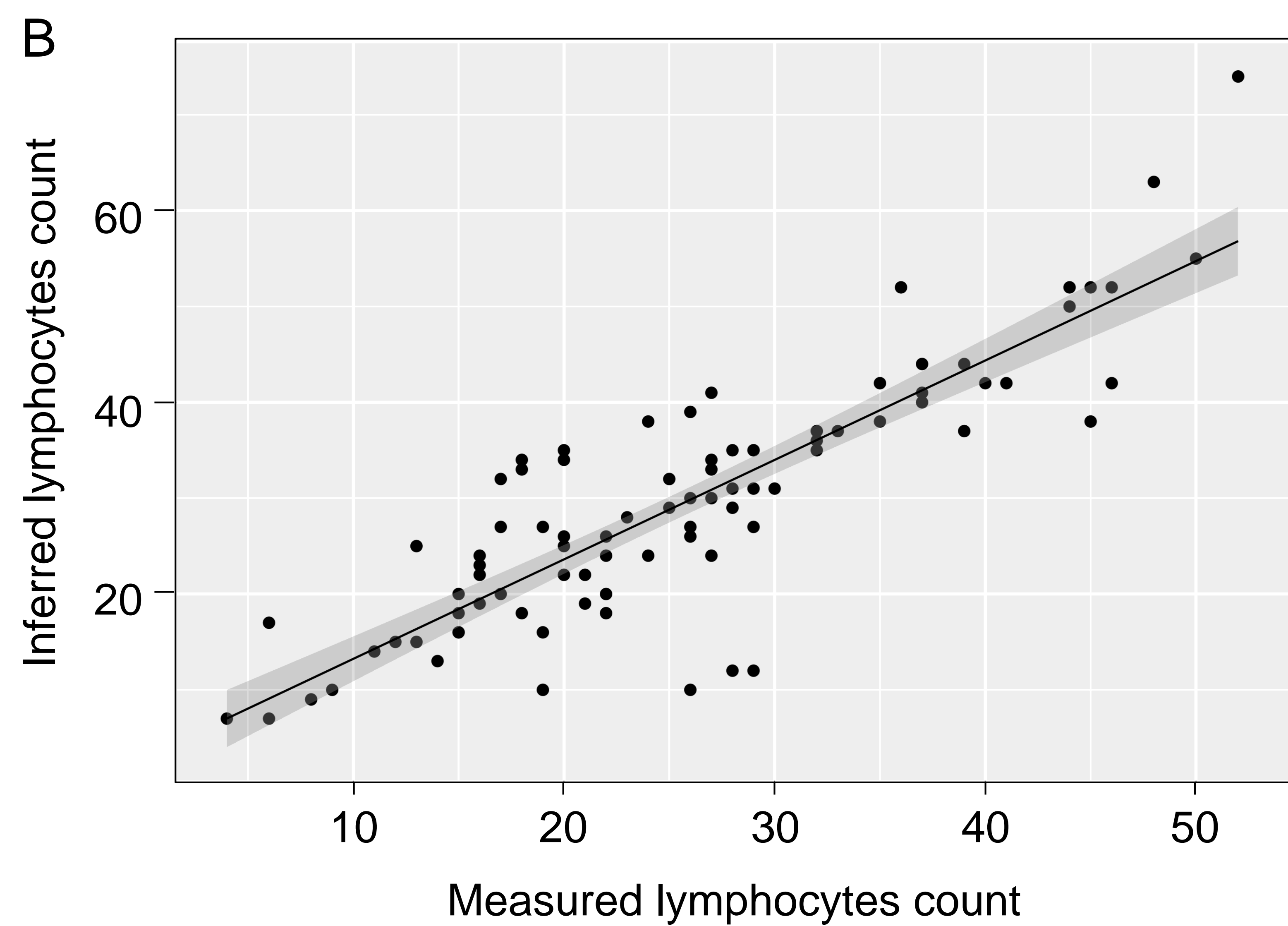

Supplement: Supplementary Figure 1 [file supplementary_figure_1.pdf]

A

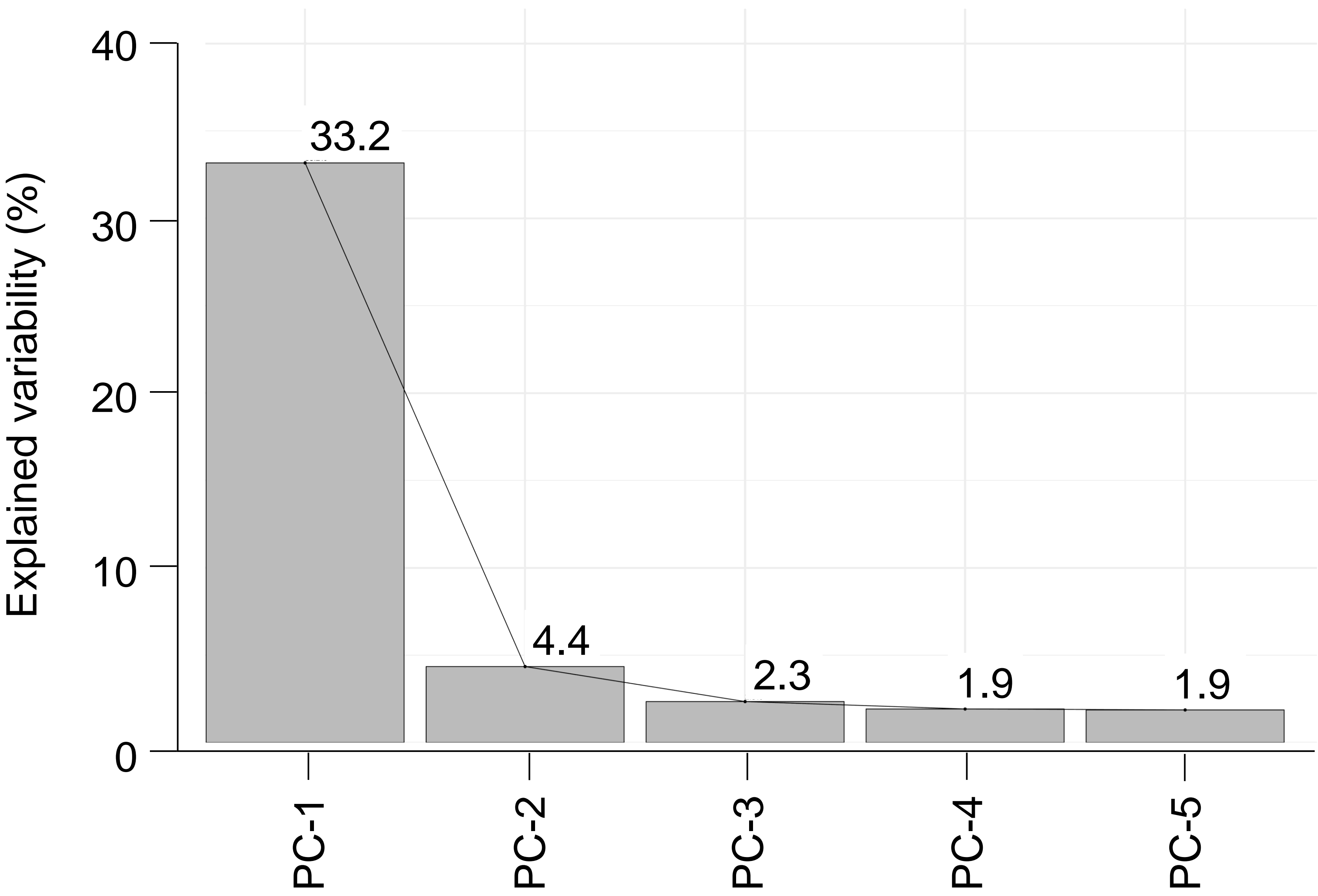

B

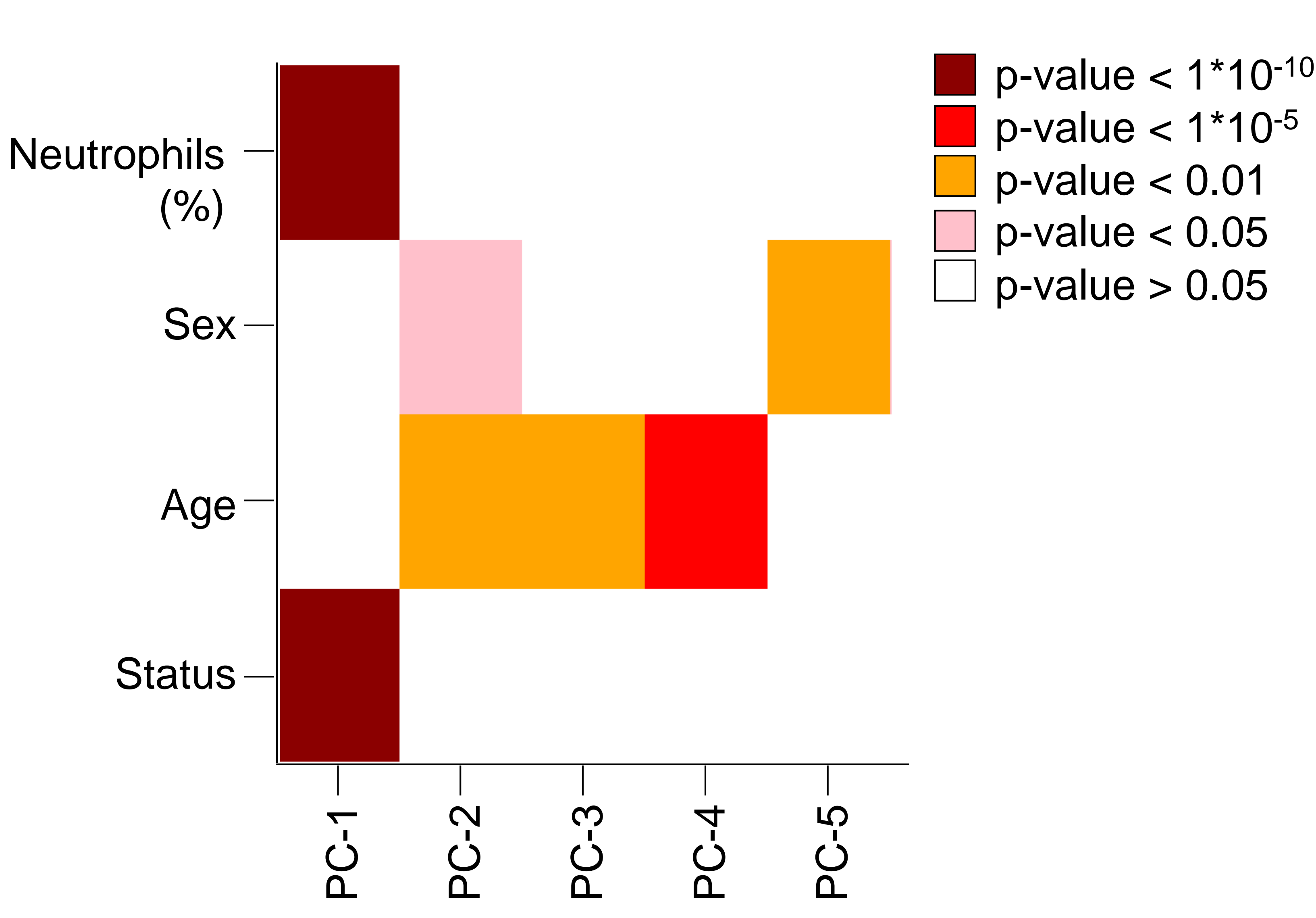

Supplement: Supplementary Figure 2 [file supplementary_figure_2.pdf]

■ Hypermethylated in overt Cushing's syndrome  
■ Hypomethylated in overt Cushing's syndrome

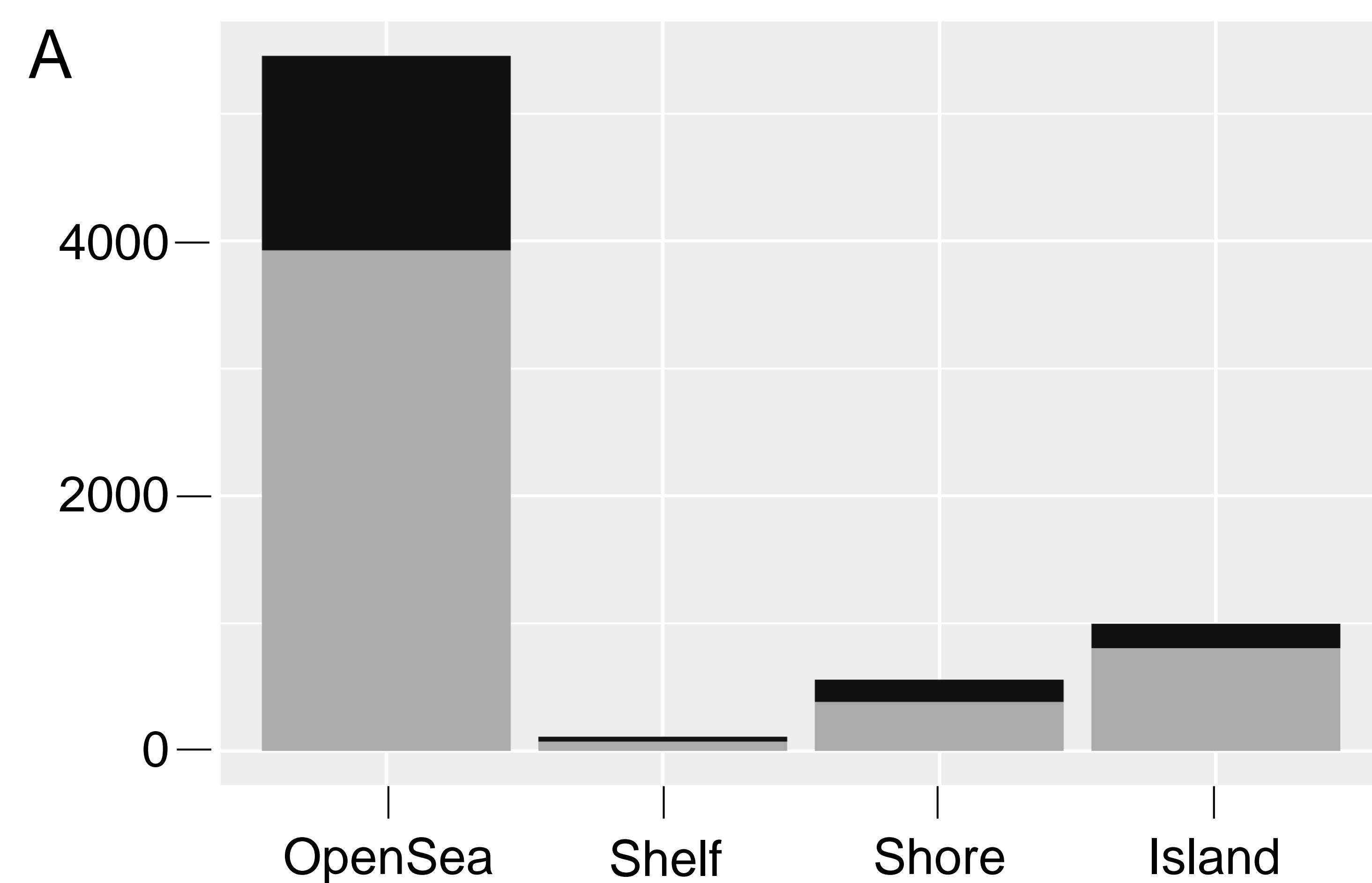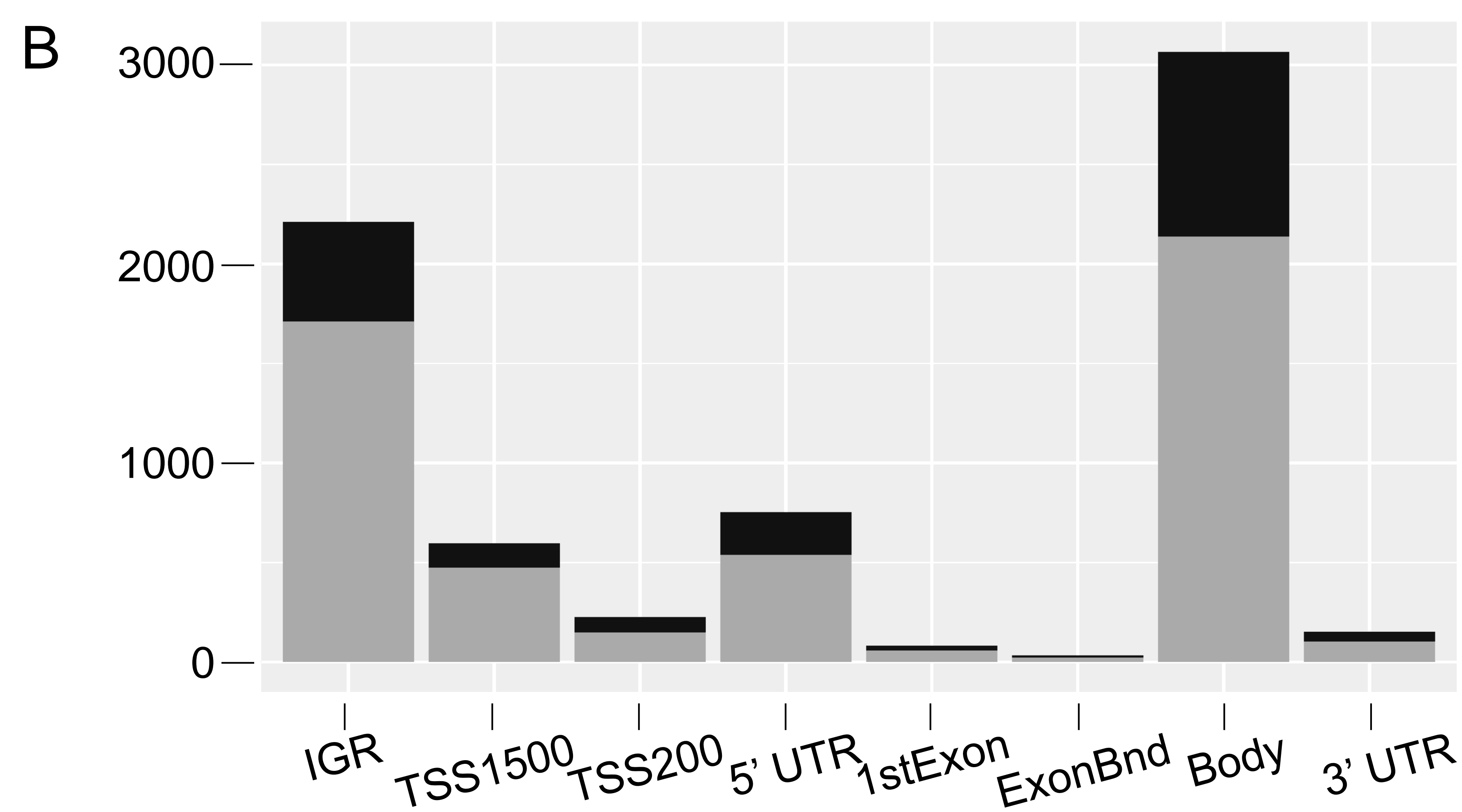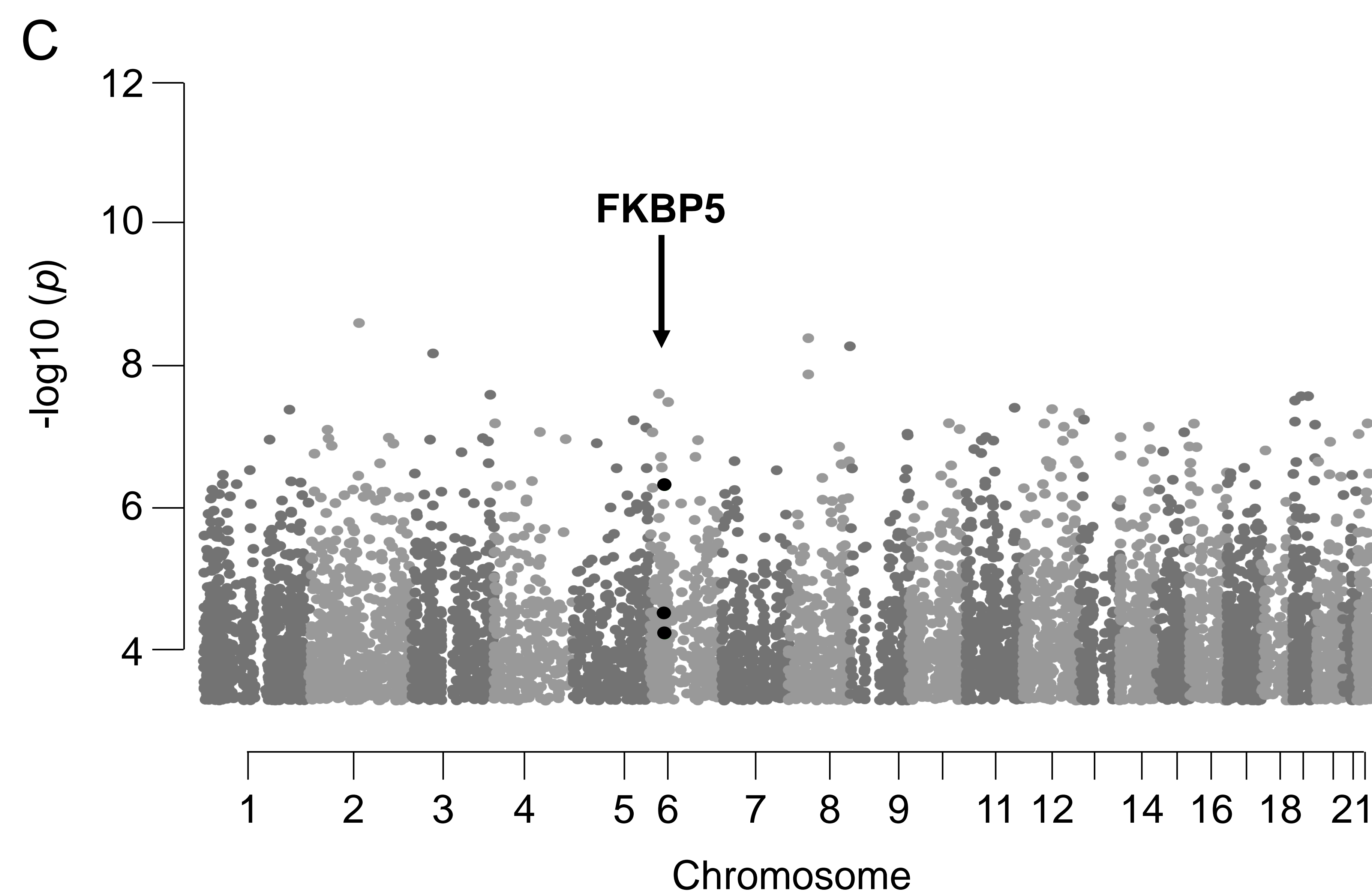

Supplement: Supplementary Figure 3 [file supplementary_figure_3.pdf]

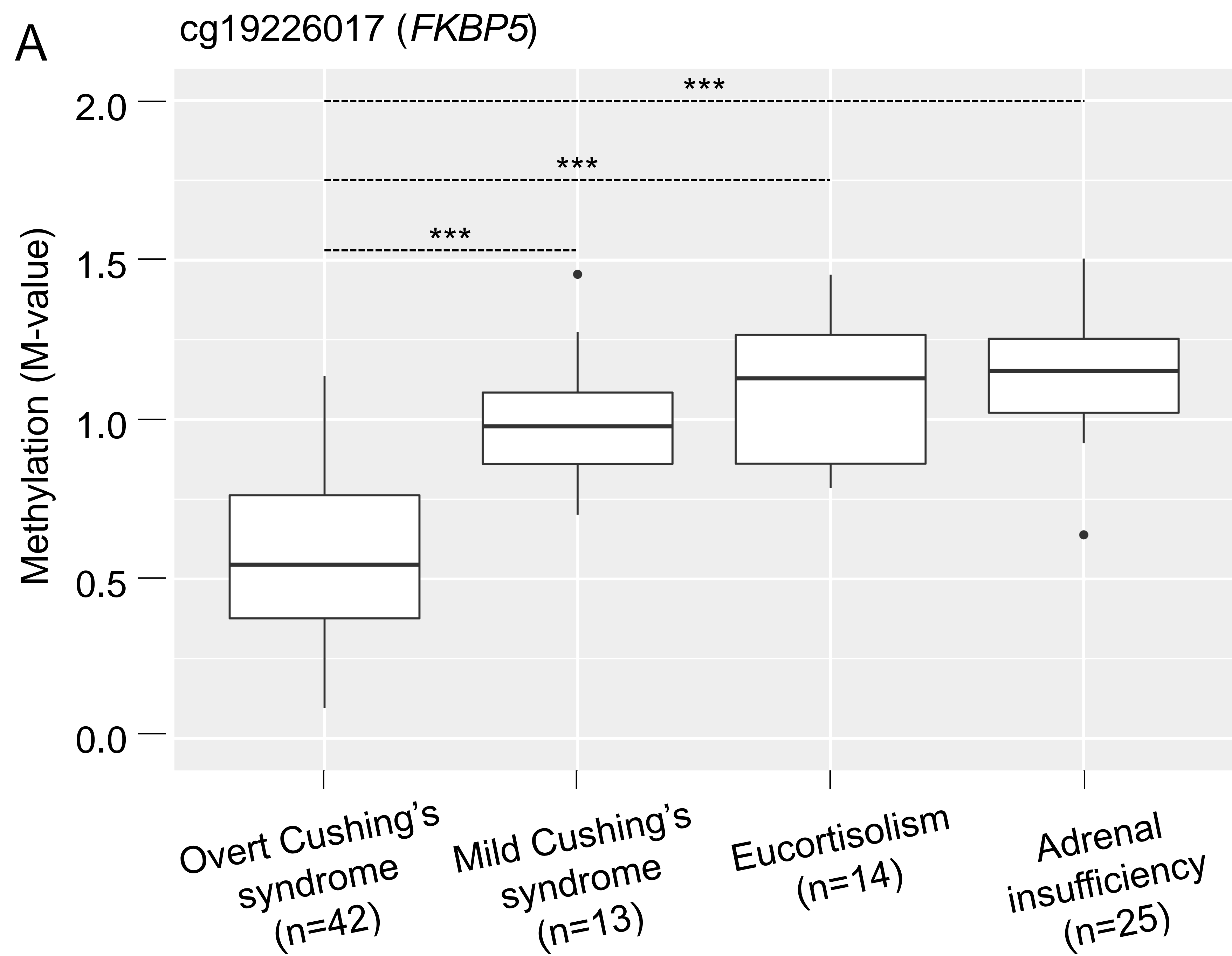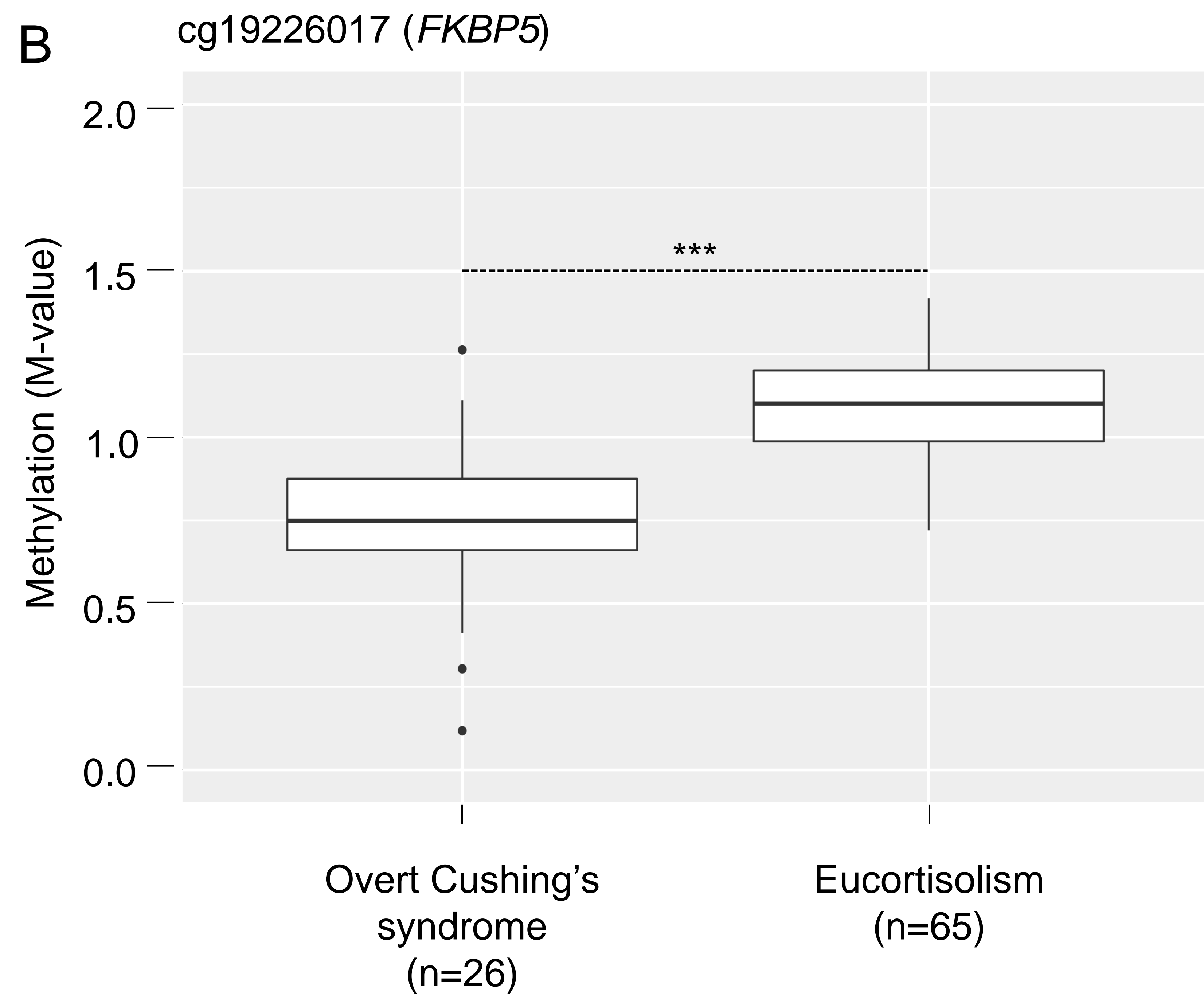

Supplement: Supplementary Figure 4 [file supplementary_figure_4.pdf]
